# Differentiating lymph node status in malignant melanoma: the role of apparent diffusion coefficient ratio in diffusion-weighted MRI – a prospective diagnostic study

**DOI:** 10.1186/s12880-026-02205-6

**Published:** 2026-02-04

**Authors:** Marc Philip Elias Kohnen, Matthias Mühler, Andreas Arnold, Stine Lutze, Chia-Jung Busch, Silvia Ribback, Kristin Peters, Marie-Luise Kromrey, Michael Kirsch, Julie Gamain, Fiona Mankertz, Carolin Malsch

**Affiliations:** 1 Institute for Diagnostic Radiology and Neuroradiology, University Medicine Greifswald, Ferdinand-Sauerbruch-Straße 1, 17475 Greifswald, Germany; 2 Clinic and Polyclinic for Skin and Venereal Diseases, University Medicine Greifswald, Ferdinand-Sauerbruch-Straße 1, 17475 Greifswald, Germany; 3 Department of Otorhinolaryngology and Head and Neck Surgery, University Medicine Greifswald, Ferdinand-Sauerbruch-Straße 1, 17475 Greifswald, Germany; 4 Institute of Pathology, University Medicine Greifswald, Friedrich-Loeffler-Str. 23e, 17487 Greifswald, Germany; 5 Institute and Polyclinic for Diagnostic and Interventional Radiology, Faculty of Medicine and University Hospital Carl Gustav Carus Dresden, TU Dresden, Fetscherstraße 74, 01307 Dresden, Germany; 6 Department for Diagnostic and Interventional Radiology, University Hospital Tübingen, Hoppe-Seyler-Straße 3, 72076 Tübingen, Germany; 7 Institute for Mathematics and Computer Science, University Greifswald, Walther-Rathenau-Str. 47, 17489 Greifswald, Germany

**Keywords:** Malignant melanoma, Lymphatic metastases, Diffusion-Weighted magnetic resonance imaging, Apparent diffusion coefficient ratio, Node-RADS

## Abstract

**Background:**

This study aimed to investigate a standardized apparent diffusion coefficient (ADC) ratio cut-off for identifying lymph node (LN) metastasis in malignant melanoma (MM), addressing the challenges of current non-invasive LN status diagnostics and dependences of absolute ADC values.

**Methods:**

This prospective, single-center study evaluated consecutive patients using diffusion-weighted MRI (DWI-MRI). We included 52 patients with early-stage MM who underwent sentinel lymph node (SLN) extraction, and 12 patients with advanced-stage MM and newly confirmed or progressive metastatic lymph nodes (MLNs). ADC values for positive and negative SLNs as well as MLNs were measured. Ratios of the ADC of SLNs or MLNs were calculated relative to benign contralateral LNs (cADC) and adjacent muscle tissue (mADC). ROC analysis identified cut-offs for absolute ADC values and relative ADC ratios. Diagnostic performance gained by regression was validated via machine learning (ML) classifiers, to evaluate robustness.

**Results:**

A total of 64 patients (median age 68.5; IQR 61–77; 46.9% female) were included. Significant differences were observed in all ADC measurements using single-shot-echo-planar-imaging (EPI): median cADC was 0.76 (IQR 0.69–0.93) for metastatic SLNs versus 1.02 (IQR 0.91–1.08) for benign SLNs (*p* = 0.03), mADC was 0.48 (IQR 0.45–0.53) versus 0.61 (IQR 0.56–0.71, *p* = 0.001). Optimal cut-offs for cADC and mADC in the SLN group were 0.81 (AUC 0.75; 95%-CI 0.47-1) and 0.49 (AUC 0.9; 95%-CI 0.78-1). These closely matched the optimal cut-offs found in the MLN set: cADC 0.83 (AUC 0.98; 95%-CI 0.96-1) and mADC 0.49 (AUC 0.99; 95%-CI 0.98-1). In both sets, these ratios outperformed morphological criteria, such as short-axis diameter (AUC 0.61; 0.38–0.84 in SLN- and 0.86; 0.8–0.92 in MLN set) and Node-RADS (AUC 0.5; 0.27–0.72 and 0.89; 0.84–0.94). ML models reproduced the diagnostic patterns observed in logistic regression.

**Conclusion:**

The introduction of a standardized ADC ratio cut-off in DW-MRI to differentiate benign from metastatic lymph nodes in MM offers a valuable, normalized, non-invasive addition to traditional diagnostics, though the findings require confirmation in larger, multicenter and multi-scanner cohorts.

**Supplementary Information:**

The online version contains supplementary material available at 10.1186/s12880-026-02205-6.

## Background

### Malignant melanoma

The escalating prevalence of malignant melanoma (MM) requires advancements in diagnostic techniques for lymphatic pathway metastasis, as lymph node (LN) status significantly impacts survival rates [1, 2]. Surgical management and adjuvant immunotherapy depend on the presence and extent of lymphatic involvement, alongside the presence of distant organ metastases, determining disease stage and guiding the treatment approach [3]. The diagnostic gold standard to this day remains surgical excision of suspicious LNs followed by histopathological analysis. Additionally, to confirm possible early-stage metastasis, radiopharmaceutical-guided sentinel lymph node excision (SLNE) represents the method of choice for assessing the status of clinically unsuspicious LNs in early-stage MM (AJCC I–II), potentially resulting in upstaging to AJCC stage III in case of a positive SLN [1, 4]. However, this invasive method requires anaesthesia, incurs possible side-effects, and is limited to early-stage melanoma. Also during follow-up, special attention is warranted to the locoregional area, as LN and in-transit metastases represent the most frequent sites of initial recurrence [5].

### Current non-invasive diagnostics

Non-invasive methods like ultrasound and CT scan, which focus on morphology and short-axis diameter (SAD), are commonly used to assess the likelihood and extent of LN metastasis. These methods show moderate performance and depend on the examiner’s experience [6]. Additionally, SAD has limited predictive value for determining LN metastasis [6–8]. The recent introduction of the Node Reporting and Data System (Node-RADS), which includes LN shape and structure as diagnostic criteria, still relies notably on subjective evaluation [9–11]. Although FDG-PET/CT imaging provides overall higher sensitivity and specificity for MM metastases, it is costly, involves relatively high radiation exposure, and is only recommended as an initial whole-body staging in higher-stage MM patients (≥ AJCC IIb [1, 6, 12–14]. Additionally, FDG-PET was considerably less sensitive for detecting locoregional LN metastases overall (sens 0.56, spec 0.97), dropping to as low (sens 0.17, spec 0.99) for LN assessment in first staging [15]. Diffusion-weighted MRI (DWI-MRI) is an alternative functional imaging modality which quantifies water molecule protons mobility, thus indicating restricted water diffusion in cellularly dense LNs [16–18].

### ADC assessment

Therefore, it is suitable for evaluating metastasized LNs by measuring the apparent diffusion coefficient (ADC). Previously, absolute ADC cut-off values have already been successfully used to assess LN metastasis in breast and cervical cancer, but a high variability among absolute ADC cut-offs limits its broader application [19, 20]. Additionally, there is a lack of published absolute ADC values specifically for metastatic LNs in cutaneous MM. Knill et al. (2023) reported median absolute ADC values of 1.42 × 10⁻³ mm²/s for metastatic LNs in advanced-stage MM, but did not provide a diagnostic cut-off [21]. Furthermore, these values clearly exceed those in breast cancer, where Luo et al. (2013) and Fardanesh et al. (2022) found ADCs of 0.787 and 0.942 × 10⁻³ mm²/s [22, 23].

In their node-to-node evaluation of initial LN staging in MM patients prior to SLNE using both MRI and FDG-PET/CT, Schaarschmidt et al. (2018) observed no significant differences in absolute ADC or tracer uptake. This may be attributed to partial-volume effects related to the ~ 34 mm³ voxel size in MRI and the low metabolic activity of typically small SLN metastases in PET/CT [12]. Currently, guidelines do not recommend absolute ADC values without reservation due to lack of validation, high variance, and inter-scanner variability [7, 24–26]. Absolute ADC values may also differ interindividually depending on tissue density, LN localization, spread of tumorous tissue, and accompanying inflammation. As such, it seems more practicable to use a diagnostic method which compares suspicious LNs to healthy tissue of the same patient. In prostate cancer, the meta-analysis by Agrotis et al. (2025) demonstrated that a ADC-ratio (prostate lesion to background prostate tissue) achieved a diagnostic performance comparable to that of the absolute ADC [27], whereas Maier et al. (2022) demonstrated that such a prostate ADC ratio can mitigate the dependence on the b-value [28]. Thus, a potential approach for LNs is to normalize their values by using a LN-to-healthy-tissue ratio—whether lymphatic or musculoskeletal—instead of relying on absolute ADC measures.

### Study objectives

So, this study aims to establish a standardized ADC ratio cut-off for differentiating benign from metastatic LNs in MMs, addressing the limitations of current non-invasive diagnostic approaches and the variability of absolute ADC values.

As a secondary methodological objective, we also assess whether the diagnostic patterns remain stable across different modelling approaches, comparing binary logistic regression with multiple machine-learning classifiers. Evaluating consistency across models with distinct assumptions provides an additional robustness check, as findings that hold both within an inferential statistical framework and across several data-driven models are generally considered more resilient. This rationale aligns with conceptual work by Bzdok et al. (2018), emphasizing complementary inference- and prediction-oriented approaches, and by Yarkoni & Westfall (2017), who highlight predictive modelling as an independent test of signal stability [29, 30].

### Hypothesis

We hypothesize that metastatic LNs exhibit significantly lower ADC values—and consequently lower intra-individual ADC ratios—than benign LNs. We further assume that these ratios offer superior diagnostic discrimination compared with conventional morphological criteria, and that the resulting cut-off is robust in both machine-learning modelling and inter-reader evaluation.

## Materials and methods

### Study design

This prospective, monocentric study was approved by the local ethics committee (reference number BB 009/23), and complied with the Helsinki Declaration, STARD [31], and STROBE guideline [32].

All participants provided informed consent. The study involved two distinct patient groups from Skin Tumor Centre at University Medicine Greifswald, Germany.

#### SLN group

**Fig. 1:**
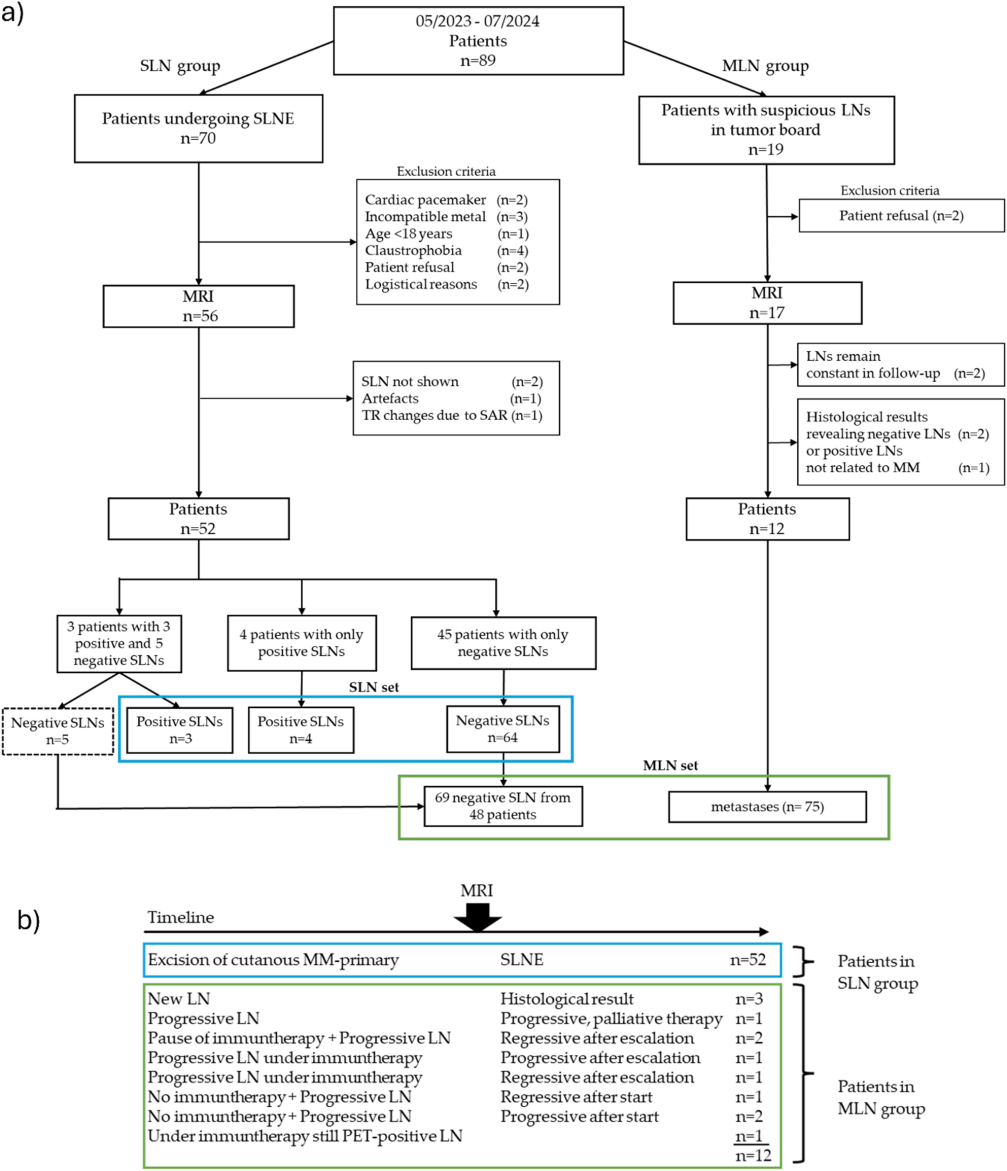
Flowchart of final included patients undergoing Sentinel lymph node extraction (SLNE) or having suspicious lymph nodes in tumor board, confirmed in follow-up (MLN). (**a**) The blue box represents the SLN set with excluded SLNs in the dashed box. The green box represents the MLN set. Definition of MLNs at MRI timepoint through dynamics in follow-up: regress or progress after start/escalation of immunotherapy. (**b**) *MM* malignant melanoma, *SAR* Specific absorption rate

Patients with newly diagnosed MM scheduled for SLNE were recruited consecutively between May 2023 and July 2024. Inclusion criteria were adults over 18, with written consent for MRI and eligibility for SLNE. Exclusion criteria included cardiac pacemakers, non-MRI-compatible implants, or claustrophobia (Fig. 1a).

#### MLN group

Between July 2023 and July 2024, patients with new or progressive lymph node metastases (MLNs), as determined by the institutional tumor board (including a senior radiologist with over 20 years of experience), Further inclusion and exclusion criteria matched those of the SLN group.

#### **Intervention and Diagnostic Standard**

SLN group patients underwent an additional MRI of regional LN stations before SLNE (0 days; ICR 0–3). SLN status was confirmed by pathology (HMB-45 antibody staining). MLN group patients underwent consecutively the same MRI protocol of affected LN regions before starting or escalating immunotherapy or excision. MLN status was defined by histopathology or by suspicious LNs at the MRI time point showing either regression under new/escalated therapy or progression despite treatment on follow-up imaging (Fig. 1b). Patients with morphologically stable LNs (indeterminate status) or histology indicating non-melanoma metastases were excluded.

### MRI protocol

Both groups underwent MRI on a 3T Magnetom Vida scanner (Siemens Healthineers, Erlangen, Germany), following a standardized protocol with anatomical and DWI sequences. The DWI protocol included transverse single-shot-spin-echo echo-planar-imaging (EPI) and readout segmentation of long variable echo-trains (RESOLVE) sequences, both set at 1.4 × 1.4 × 3 mm, with b-values of 50 and 800 s/mm², four-directional trace-weighted-imaging, and strong spectral fat suppression. The total acquisition time was approximately 7 min each (details in suppl. Table 1). ADC maps were generated by the Syngo.via VB60 software (Siemens Healthineers, Munich, Germany).

Anatomical sequences included transverse T1-weighted (TR 552 ms, TE 17 ms, 0.4 × 0.4 × 3 mm³) and T2-weighted Dixon (TR 3650 ms, TE 56 ms, 0.5 × 0.5 × 3 mm). BioMatrix coils (Spine 72 WS, Head/Neck 20, and Body 18, Siemens Healthineers, Erlangen, Germany) ensured signal acquisition.

### Imaging analysis

SLN localization involved intradermal injection of a radiopharmaceutical, followed by SPECT-CT imaging (NM/CT 870 DR, GE HealthCare, Chicago, Illinois, USA) to determine SLN positions before surgery. Extracted SLNs were immediately sent for histopathological analysis. Imaging data were collected by a first reader (R1) and supervised by a senior radiologist and nuclear medicine physician. All were blinded to the histopathological results.

**Fig. 2:**
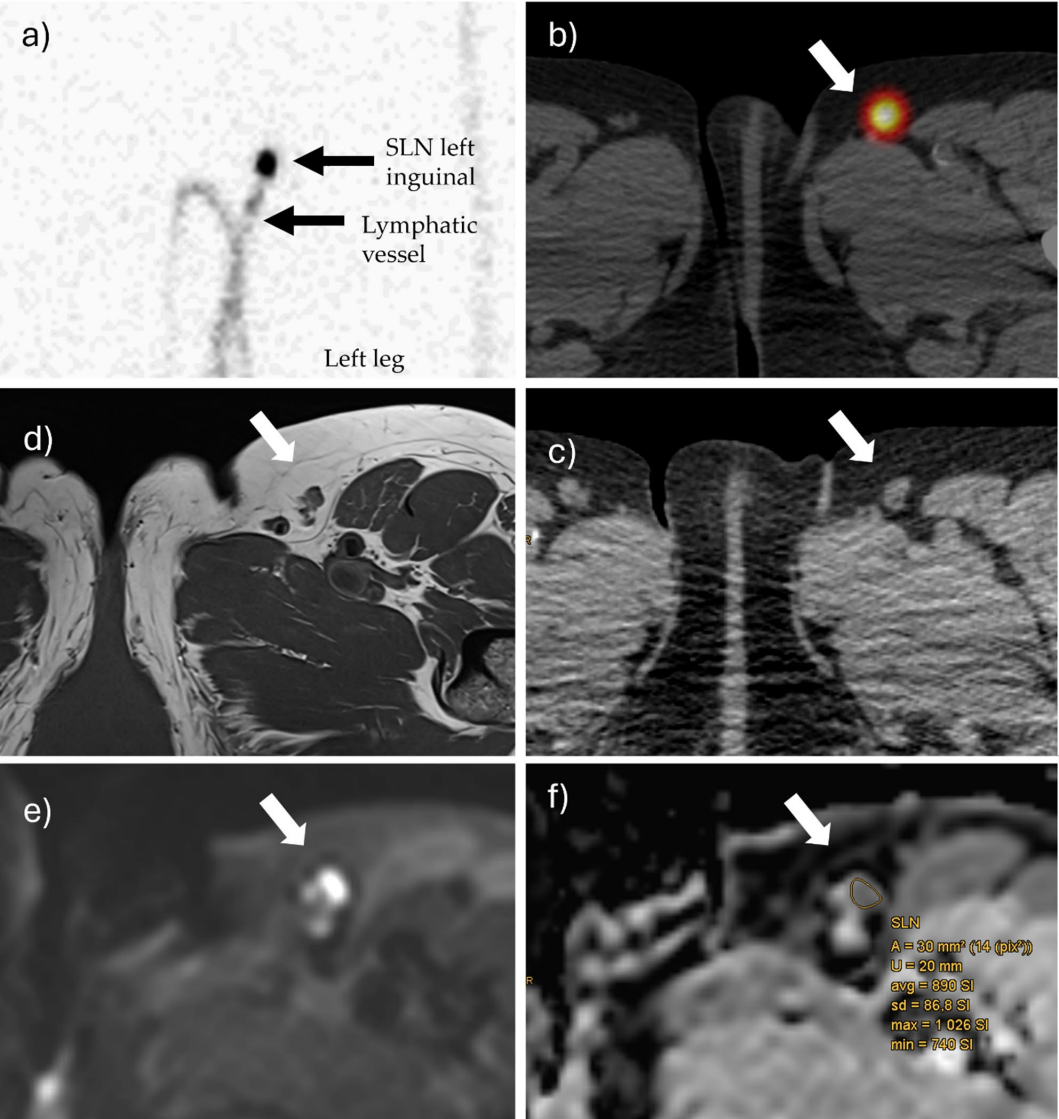
Data acquisition workflow. Mapping of the SLN in the static scintigraphy after injection of ^99m^Tc-labeled colloids around the MM-resection scar (left leg). The tracer drains via a lymphatic vessel to the first lymph node, the SLN (**a**). Three-dimensional imaging using SPECT-CT (**b**). Identification of the SLN (white arrows) in the low-dose-CT component (**c**) and its corresponding location in the T1w-sequence of MRI (**d**) and b800-sequence of DWI-MRI (**e**). ROI placement based on hyperintensity in b800 and corresponding hypointensity in the ADC map (**f**). ADC measurements were also performed for three contralateral lymph nodes and adjacent muscle tissue to calculate cADC and mADC. *cADC* Ratio of LN ADC to mean ADC of 3 contralateral LNs, *mADC* Ratio of LN ADC to ADC of ipsilateral muscle *MM* malignant melanoma, *SLN* Sentinel lymph node, *SPECT* Single Photon Emission Computed Tomography

A two-dimensional Region of interest (ROIs) were manually drawn on the ADC map in the LNs’ parenchyma (see Fig. 2) for both DWI sequences, focusing on areas with the lowest ADC signal (and high b800 signal) according to established mpMRI guidelines for breast and prostate lesions [24, 33]. Artifacts, hilum, fat tissue, cystic/necrotic regions, and regions falsely appearing hypointense due to partial volume effects from adjacent suppressed fat tissue were excluded. Each ROI covered more than one pixel to ensure reliability. Mean ADC values were recorded (10⁻⁶ mm²/s).

Volume of interest (VOI) ADC values were manually measured across as much LN parenchyma as possible in each slice following the same rules using mint Lesion software (version 3.8.6, Mint Medical GmbH, Heidelberg, Germany).

VOI (EPI) and ROI-based ADC values (EPI and RESOLVE) were also measured for three randomly selected contralateral non-malignant LNs from the same patient. The ratio ‘cADC’ was defined as the ADC of the target LN (tLN) relative to the mean ADC of three contralateral LNs (cLN). The ‘mADC’ ratio was calculated as the tLN’s ADC relative to the ADC of a fixed 20 mm diameter ROI in adjacent muscle (M).

$$cADC = {\rm{ }}AD{C_{tLN}}/{\rm{ }}\left( {\left( {AD{C_{cLN1}} + {\rm{ }}AD{C_{cLN2}} + {\rm{ }}AD{C_{cLN3}}} \right){\rm{ }}/3} \right)$$

$$mADC{\rm{ }} = {\rm{ }}AD{C_{tLN}}/{\rm{ }}AD{C_{M}}$$

All LNs were additionally evaluated using Node-RADS criteria [9] and measured for short-axis diameter (SAD) in mm. All ROI-based ADC assessments in both EPI and RESOLVE for SLNs and MLNs as well as their contralateral LNs, the reference muscle, short-axis diameter (SAD), and Node-RADS were independently performed by a second blinded reader (R2) with three years of experience.

### Statistical methods

To assess similarities and differences between patients with metastatic and benign LNs, we compared patient characteristics and imaging-derived metrics as shown in Table 1. Categorical variables were summarized as frequencies, and continuous variables as median and quartiles. Differences between groups were tested with Fisher’s exact or Chi-square tests for categorical variables and t-tests or Mann-Whitney U tests for continuous variables, depending on distributional assumptions. A *p*-value < 0.05 was considered significant. There was no missing data.

Main analysis: Receiver operating characteristic (ROC) analysis was used to assess the diagnostic performance of ADC parameters by determining the optimal ADC cut-offs. The ROC curve plots the true positive rate (sensitivity) against the false positive rate (1 – specificity) across varying thresholds. The area under the ROC curve (AUC) quantifies the overall discriminative ability of a parameter, ranging from 0.5 (no discrimination) to 1.0 (perfect discrimination). Youden’s index was used to find the best cut-off. Agreement with LN status was evaluated using Gwet’s AC1 coefficient [34, 35]. Correlations between ROI size and ADC values were assessed using Spearman’s rank correlation coefficient (rho = ρ).

Validation: Five commonly used machine learning (ML) algorithms representing different model families (tree-based, boosting, distance-based, probabilistic, and neural approaches) were selected to validate the robustness of logistic-regression-based performance estimates: Random Forest [36], Gradient Boosting (XGBoost) [37], K-Nearest Neighbors (KNN) [38], Naive Bayes [39], and Neural Nets [40], assessing performance metrics. A complete list of all model specifications, including the default and study-specific hyperparameters used for each machine-learning algorithm, is provided in the Supplementary Text 1.

Diagnostic performance metrics including AUC with 95% confidence intervals, accuracy, sensitivity, specificity, PPV, NPV, Youden’s index, and optimal cut-off were derived from the confusion matrix (true positives [TP], false positives [FP], true negatives [TN], false negatives [FN], see suppl. formulas 1) and evaluated for each method. Sensitivity (TP / [TP + FN]) reflects how well a test identifies patients with metastasized LNs and is crucial in screening setting to reduce FNs. Specificity (TN / [TN + FP]) indicates how well a test identifies patients with benign LNs and is essential in confirmatory diagnostics to reduce FPs. Therefore, sensitivity and specificity represent a key diagnostic metric in our study. Youden’s index (sensitivity + specificity – 1) was used to determine the optimal cut-off for each ADC parameter. It provides a single summary measure that maximizes the combined sensitivity and specificity of a test.

Reliability Analysis: Agreement between the two readers across all measured LNs was evaluated using the intraclass correlation coefficient (ICC 2,1, single measures, absolute agreement [41] with corresponding 95% confidence intervals, and further analyzed by Bland–Altman statistics (bias and 95% limits of agreement) [42]. In addition, the optimal ADC ratio cut-off derived from SLN-set (Reader 1) was evaluated on the measurements of all LNs obtained by Reader 2 to assess its validity.

Statistical evaluations were performed by a clinical scientist using Microsoft Excel (version 2502, Microsoft Corporation, Redmond, Washington, USA) and SPSS (version 29, IBM, Armonk, New York, USA). Independent validation and ML approaches were conducted by a statistician using R software (version R 4.4.3, The R Foundation, Vienna, Austria).

Bias: Selection bias was minimized by consecutive recruitment, observer bias by blinding, and information bias by standardized DWI protocols. Histological results were clearly matched to SLNs via SPECT-CT.

## Results

### Final samples

To ensure clarity, we distinguish between patient-based cohorts (“groups”) and LN-based analysis datasets (“sets”).

SLN group: Of 70 initially screened patients, 18 were excluded, resulting in 52 patients in the SLN group, contributing 76 SLNs with histopathological results. The target sample size (71), based on power and agreement with LN status, was reached (for assumptions see Supplementary Text 2). 45 patients had only negative SLNs (*n* = 64), four had only positive SLNs (*n* = 4), and three had both negative (*n* = 5) and positive SLNs (*n* = 3).

MLN group: Due to the small number of positive SLNs in the SLN group (7/71), a second group (MLN group) was recruited, consisting of 12 patients with 75 positive LNs (Fig. 1a, b).

SLN set: After excluding five negative SLNs from patients with positive SLNs to avoid binding effects, 71 evaluable SLNs remained for analysis (7 metastatic, 64 benign).

MLN set: Pooled data from 75 positive LNs in the MLN group and 69 negative LNs from the SLN group.

### Patient characteristics

#### SLN patients

Of 52 MM patients, 26 (50%) were female, with a median age of 66.5 years (IQR 60.5–75.5). The most common subtype was superficial spreading melanoma (*n* = 26, 50%), with 23 patients (44%) at AJCC stage Ib (Table 1, and suppl. Table 2). The median thickness of the cutaneous primary was 1.85 mm (IQR 1.3–3.35). Primarily SLN localization was inguinal (*n* = 35, 49.3%). Histopathology revealed 64 benign SLNs (90.1%) and 7 metastatic SLNs (9.9%), two of which contained micrometastases (1 and 1.5 mm).

#### MLN patients

Of the MLN group of 12 patients, 4 (33.3%) were female, with a median age of 73 years (IQR 66.25–77.5). The most common subtype was nodular (*n* = 5, 41.7%), predominantly at AJCC stage IV (*n* = 10, 83%). The trunk was the most frequent primary tumor location (*n* = 4, 33.3%), including some cases with unknown primary location (CUP, *n* = 3, 25%). Most LNs (*n* = 44, 58.7%) were located in the axillary basin (Table 3, suppl. Table 3). The median time between the initial diagnosis of cutaneous MM and (re-)presentation in tumor board/MRI was 31 months (IQR 1–48).

### SLN set: imaging analysis

#### Absolute ADC values and morphology

**Fig. 3:**
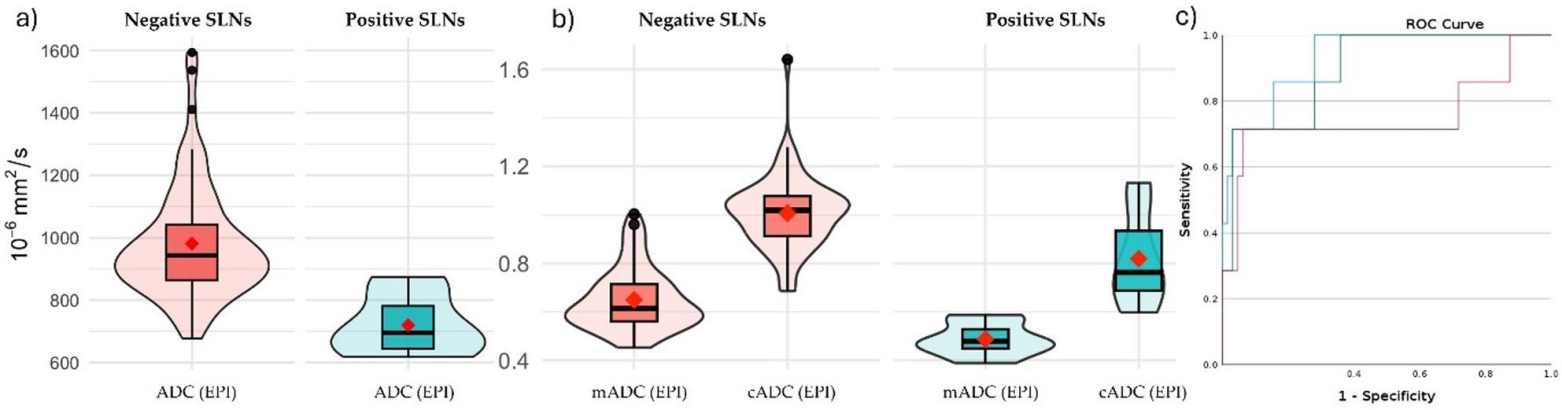
Violin-plots for absolute ADC-values (**a**) and as a ratio (**b**) to contralateral lymph nodes (cADC) and adjacent muscle (mADC) in EPI-sequence for negative (red) and positive SLNs (turquoise) in SLN set. Corresponding ROC curves of ADC (blue line), cADC (red line) and mADC (green line) in EPI-Sequence (**c**) *SLN* Sentinel lymph node, *EPI* Single shot spinecho echoplanar imaging

ROI-based median ADC values of metastatic SLNs were significantly lower than benign LNs in both EPI (*p* < 0.001) and RESOLVE (*p* = 0.002, Table 1). E.g., the median ADC was 695 (IQR 644-781.5) × 10⁻⁶ mm²/s for metastatic SLNs, vs. 944 (IQR 864.5-1042.75) × 10⁻⁶ mm²/s for benign SLNs in EPI (Fig. 3a). No significant difference was found for volumetric ADC (*p* = 0.17), SAD (7 (IQR 5.5–7.5) mm vs. 5 (IQR 4–7) mm, *p* = 0.34) or Node-RADS (*p* = 0.98). The ROI size in positive SLNs was smaller than in negative SLNs in both EPI and RESOLVE sequences, whereas the voxel size in relation to LN volume remained constant (Table 1). Additionally, there was no significant correlation between ADC and ROI size (EPI: ρ = 0.137, *p* = 0.256, RES: ρ = 0.171, *p* = 0.154, Vol: ρ = 0.138, *p* = 0.252). ROC analysis yielded an AUC of 0.93 (95%-CI 0.84-1) for absolute ADC in EPI (cut-off 847 × 10⁻⁶ mm²/s, sensitivity 1, specificity 0.72).

**Table 1:** Patients characteristics, ADC metrics and morphology in SLN set. In brackets interquartile range (IQR) or percentages (%)

Variable	Overall	Positive	Negative	*p*
Patients	52	7	45	
Age (y; median, IQR)	66.50 (60.5, 75.5)	66.00 (60.5, 80)	67.00 (61, 75)	0.788
Male (n; %)	26 (50)	4 (57.1)	22 (48.9)	0.999
Female (n; %)	26 (50)	3 (42.9)	23 (51.1)	
**Sentinel Lymph Nodes**	**71**	**7**	**64**	
Axillary (n; %)	32 (45.1)	3 (42.9)	29 (45.3)	0.575
Cervical (n; %)	4 (5.6)	1 (14.3)	3 (4.7)	
Inguinal (n; %)	35 (49.3)	3 (42.9)	32 (50)	
**Absolute ADC**				
EPI (10⁻⁶ mm²/s; median, IQR)	917 (845.5, 1028.5)	695 (644, 781.5)	944 (864.5, 1042.75)	< 0.001
RESOLVE (10⁻⁶ mm²/s; median, IQR)	948 (839, 1103)	799 (715, 873)	982 (853.5, 1107)	0.002
Volumetry (10⁻⁶ mm²/s; median, IQR)	1164 (983, 1424)	1020 (895, 1187)	1173.5 (983.75, 1429)	0.171
**Ratio (cADC)**				
EPI (median, IQR)	1.01 (0.90, 1.07)	0.76 (0.69, 0.93)	1.02 (0.91, 1.08)	0.031
RESOLVE (median, IQR)	0.94 (0.84, 1.10)	0.77 (0.69, 1.01)	0.95 (0.85, 1.10)	0.097
Volumetry (median, IQR)	0.98 (0.87, 1.09)	0.95 (0.74, 1.03)	0.99 (0.88, 1.09)	0.316
**Ratio (mADC)**				
EPI (median, IQR)	0.61 (0.55, 0.71)	0.48 (0.45, 0.53)	0.61 (0.56, 0.71)	0.001
RESOLVE (median, IQR)	0.57 (0.52, 0.65)	0.49 (0.43, 0.54)	0.59 (0.52, 0.65)	0.010
Volumetry (median, IQR)	0.76 (0.64, 0.95)	0.70 (0.63, 0.81)	0.78 (0.65, 0.98)	0.316
**ROI/VOI sizes**				
VOI (mm^3^)	450 (180, 890)	250 (160, 730)	460 (192.5, 997.5)	0.255
ROI EPI (Voxel)	9 (6, 14)	4 (3, 6)	10 (7, 14)	< 0.001
ROI EPI (Vox/VOI)	0.12 (0.07, 0.23)	0.09 (0.05, 0.14)	0.13 (0.07, 0.23)	0.263
ROI RES (Vox)	9 (6, 11)	3 (3, 5)	9 (7, 12)	< 0.001
ROI RES (Vox/VOI)	0.06 (0.11, 0.22)	0.07 (0.04, 0.14)	0.11 (0.07, 0.24)	0.162
**Morphology**				
Node-RADS (median, IQR)	2 (1, 2)	2 (1, 2)	2 (1, 2.25)	0.983
SAD (mm; median, IQR)	6 (4.5, 7)	7 (5.5, 7.5)	5 (4, 7)	0.344

*ADC* Apparent diffusion coefficient, *cADC* Ratio of LN ADC to mean ADC of 3 contralateral LNs, *EPI* Single shot spinecho echoplanar imaging, *mADC* Ratio of LN ADC to ADC of ipsilateral muscle, *Node-RADS* Node Reporting and Data System, *RESOLVE/RES* Readout segmentation of long variable echo trains sequence, *ROI* Region of interest *SAD* Short-axis diameter, *SLN* Sentinel lymph node, *VOI* volume of interest *Volumetry* Volumetry measuring, *Vox* Voxel

#### ADC ratios

Both ROI-based ratios in EPI significantly differed between metastatic and benign SLNs (Table 1; Fig. 3b). The median mADC was 0.48 (IQR 0.45–0.53) vs. 0.61 (IQR 0.56–0.71, *p* = 0.001), and cADC 0.76 (IQR 0.69–0.93) vs. 1.02 (IQR 0.91–1.08, *p* = 0.03).

**Fig. 4:**
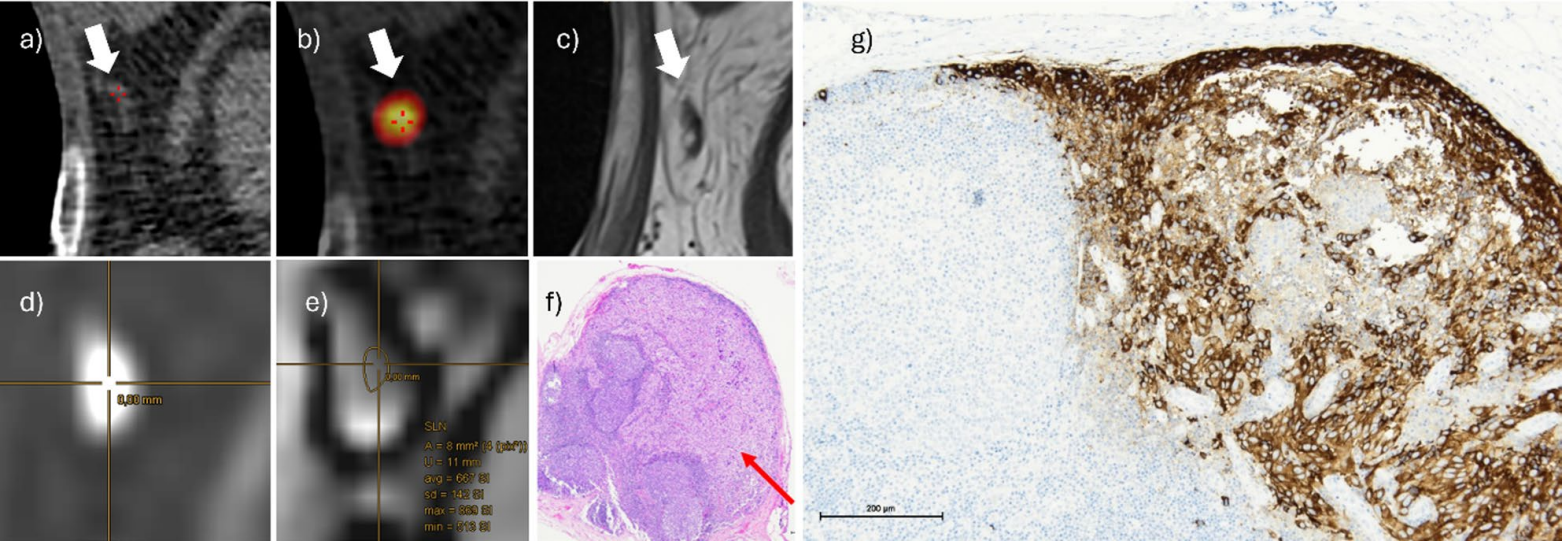
Study patient with malignant melanoma on the left breast. Preoperative SPECT/CT following ^99m^Tc-marked colloid injection circumferentially around the resection scar, demonstrating SLN localization in the left axillary basin. (**a** and **b**) Identification of the SLN in T1w (**c**, white arrows). ROI placement based on hyperintensity in b800 (**d**) and corresponding hypointensity in the ADC (**e**) The corresponding cADC was 0.66, and mADC 0.46 (cut-off 0.83 and 0.49. *The lower the ratio*,* the higher the probability of a malignant lesion*). SLN infiltration (red arrow) in H&E staining (**f**) HMB-45 antibody staining confirming the presence of melanoma cells (**g**)

Figure 4 shows an example of a metastasized SLN. The related optimal cut-off was 0.49 (AUC 0.9; 95%-CI 0.78-1; sensitivity 0.71, specificity 0.97) for mADC, and 0.81 (AUC 0.75; 95%-CI 0.47-1; sensitivity 0.71, specificity 0.94) for cADC (Fig. 3c; Table 2) with a false negative rate of 2/7 (0.29) each. Both false-negative SLNs contained micrometases. Ratios in volumetry and cADC in RESOLVE showed no significant differences. For the second reader as well, ADC (EPI), cADC (EPI), and mADC (EPI) were significantly lower in positive SLNs, whereas the remaining parameters showed no significant differences (see suppl. Table 4).

**Table 2:** Performance of ROC curve analysis within the optimized cut-off in SLN set

Variable	Cut-off	AUC	CI_l_	CI_u_	Acc	Sens	Spec	PPV	NPV	*R* ^2^
ADC (EPI) (10⁻⁶ mm²/s)	874	0.93*	0.84	1**	0.75	1**	0.72	0.28	1**	0.47
ADC (RES) (10⁻⁶ mm²/s)	925	0.85	0.73	0.97*	0.69	1**	0.66	0.24	1**	0.24
ADC (VOL) (10⁻⁶ mm²/s)	1111	0.66	0.43	0.89	0.61	0.71	0.59	0.16	0.95*	0.05
cADC (EPI)	0.81	0.75	0.47	1**	0.92*	0.71	0.94*	0.56	0.97*	0.2
cADC (RES)	0.77	0.69	0.42	0.96*	0.89	0.57	0.92*	0.44	0.95*	0.09
cADC (VOL)	0.81	0.62	0.35	0.88	0.83	0.43	0.88	0.27	0.93*	0.02
mADC (EPI)	0.49	0.9*	0.78	1**	0.94*	0.71	0.97*	0.71	0.97*	0.37
mADC (RES)	0.6	0.8	0.64	0.96*	0.52	1**	0.47	0.17	1**	0.18
mADC (VOL)	0.74	0.62	0.41	0.83	0.58	0.71	0.56	0.15	0.95*	0.04
SAD (mm)	6	0.61	0.38	0.84	0.54	0.71	0.52	0.14	0.94*	0.01
Node-RADS	5	0.5	0.27	0.72	0.92*	0.14	1**	1**	0.91*	0.01

*value > 0.9, **value = 1

*Acc* Accuracy, *AUC* Area under the curve, *ADC* Apparent diffusion coefficients, *cADC* Ratio of LN ADC to mean ADC of 3 contralateral LNs, *CI*_*l*_ Lower confidence interval, *CI*_*u*_ Upper confidence interval, *EPI* Single shot spinecho echoplanar imaging, *mADC* Ratio of LN ADC to ADC of ipsilateral muscle, *Node-RADS* Node Reporting and Data System, *NPV* Negative predictive value, *PPV* Positive predictive value, *R*^*2*^ McFadden Index, *RES* Readout segmentation of long variable echo trains sequence (RESOLVE), *SAD* Short-axis diameter, *Sens* Sensitivity, *SLN* Sentinel lymph node, *Spec* Specificity, *VOL* Volumetry measuring

### MLN set: imaging analysis

#### Absolute ADC values and morphology

**Fig. 5:**
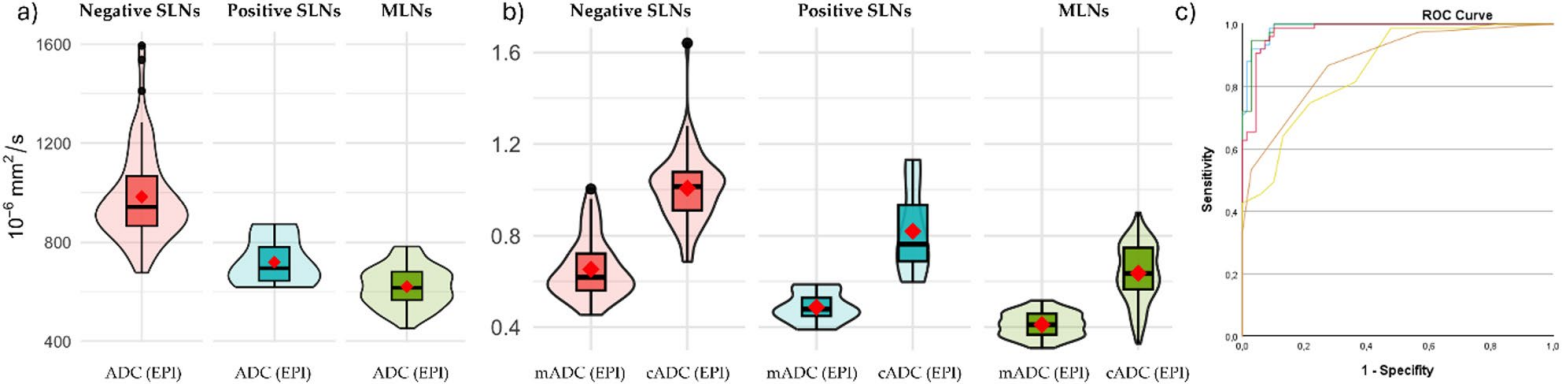
Violin-plots for absolute ADC-values (**a**) and as a ratio (**b**) to contralateral lymph nodes (cADC) and adjacent muscle (mADC) in EPI-sequence for negative LNs (red) and confirmed metastases (MLNs, olive) in MLN-set. For orientation, positive SLNs from the SLN set (turquoise) are integrated centrally. Corresponding ROC curves of absolute ADC (blue line), cADC (red line), mADC (green line), Node-RADS (brown) and short-axis diameter in mm (yellow) in MLN set (**c**). *EPI* Single shot spinecho echoplanar imaging, *SLN* Sentinel lymph node

Median ADC values of MLNs were significantly lower in all measurements (EPI and RESOLVE, Table 3; Fig. 5a); for example, 616 (IQR 566–681) × 10⁻⁶ mm²/s vs. 943 (IQR 866–1066) × 10⁻⁶ mm²/s in EPI (*p* < 0.001). There was no significant difference in ROI and VOI sizes between MLNs and benign LNs.

Node-RADS scores were significantly higher in MLNs (median 4, IQR 3–5) than in benign LNs (median 2, IQR 1–3, *p* < 0.001). Similarly, the median short-axis diameter was greater in MLNs, measuring 9 mm (IQR 7-15.5) compared to 5 mm (IQR 4–7, *p* < 0.001). ROC analysis identified optimal cut-offs at 781 × 10⁻⁶ mm²/s for ADC in EPI (AUC 0.99; 95%-CI 0.98-1, sensitivity 0.99, specificity 0.91) and at a Node-RADS score of 3 (AUC 0.89; 95%-CI 0.84–0.94, sensitivity 0.87, specificity 0.74).

**Table 3:** Patients characteristics, ADC metrics and morphology in MLN set. In brackets interquartile range (IQR) or percentages (%)

Variable	Overall	MLN group	Negative group*	*p*-value
**Patients**	**60**	**12**	**48**	
Age (y; median, IQR)	68.5 (61,77.5)	73 (66.25, 77.50)	67 (59.5, 76.25)	0.426
Male (n; %)	31 (51.7)	8 (66.7)	23 (47.9)	0.401
Female (n; %)	29 (48.3)	4 (33.3)	25 (52.1)	
**Lymph nodes**	**144**	**75**	**69**	
Axillary (n; %)	73 (50.7)	44 (58.7)	29 (42.0)	< 0.001
Cervical (n; %)	8 (5.6)	2 (2.7)	6 (8.7)	
Iliacal (n; %)	2 (1.4)	2 (2.7)	0 (0.0)	
Inguinal (n; %)	41 (28.5)	7 (9.3)	34 (49.3)	
Mesenterial (n; %)	20 (13.9)	20 (26.7)	0 (0.0)	
**Absolute ADC**				
EPI (10⁻⁶ mm²/s; median, IQR)	743 (611.75, 929.25)	616 (566, 681)	943 (866, 1066)	< 0.001
RESOLVE (10⁻⁶ mm²/s; median, IQR)	857 (757.75, 1042.5)	762 (692.50, 869.5)	990 (858, 1111)	< 0.001
Volumetry (10⁻⁶ mm²/s; median, IQR)	879.5 (761.75, 1166.25)	771 (695, 815)	1174 (998, 1421)	< 0.001
**Ratio (cADC)**				
EPI (median, IQR)	0.78 (0.63, 1.01)	0.63 (0.56, 0.75)	1.01 (0.91, 1.08)	< 0.001
RESOLVE (median, IQR)	0.85 (0.67, 0.99)	0.70 (0.51, 0.86)	0.96 (0.85, 1.10)	< 0.001
Volumetry (median, IQR)	0.81 (0.63, 1.00)	0.65 (0.51, 0.74)	0.98 (0.88, 1.09)	< 0.001
**Ratio (mADC)**				
EPI (median, IQR)	0.48 (0.41, 0.61)	0.41 (0.37, 0.46)	0.62 (0.56, 0.72)	< 0.001
RESOLVE (median, IQR)	0.52 (0.45, 0.62)	0.46 (0.41, 0.52)	0.61 (0.53, 0.67)	< 0.001
Volumetry (median, IQR)	0.58 (0.50, 0.76)	0.51 (0.46, 0.54)	0.78 (0.66, 0.92)	< 0.001
**ROI/VOI sizes**				
VOI (mm^3^)	440 (212.5, 1070)	480 (220, 1270)	380 (185, 925)	0.249
ROI EPI (Voxel)	8 (6, 13)	7 (4, 12)	10 (6, 14)	0.06
ROI RES (Voxel)	9.5 (6., 14.75)	10 (6, 17)	9 (6, 12)	0.155
**Morphology**				
SAD (mm; median, IQR)	7 (5, 11)	9 (7.5, 15.5)	5 (4, 7)	< 0.001
Node-RADS (median, IQR)	3 (2, 4)	4 (3, 5)	2 (1, 3)	< 0.001

*all negative LNs from SLN-patients

*ADC* Apparent diffusion coefficient, *cADC* Ratio of LN ADC to mean ADC of 3 contralateral LNs, *EPI* Single shot spinecho echoplanar imaging, *mADC* Ratio of LN ADC to ADC of ipsilateral muscle, *MLN* Confirmed metastasized lymph node, *Node-RADS* Node Reporting and Data System, *RESOLVE* Readout segmentation of long variable echo trains sequence, *ROI* Region of interest, *SAD* Short-axis diameter, *SLN* Sentinel lymph node, *VOI* volume of interest *Volumetry* Volumetry measuring

#### ADC ratios

All calculated ratios (in EPI or RESOLVE, and ROI or VOI) were significantly lower in MLNs compared to benign LNs (*p* < 0.001, Table 3; Fig. 5b); for example, 0.63 (IQR 0.56–0.75) vs. 1.01 (IQR 0.91–1.08, *p* < 0.001) for cADC (EPI) and 0.41 (IQR 0.37–0.46) vs. 0.62 (IQR 0.56–0.72, *p* < 0.001) for mADC (EPI).

**Fig. 6:**
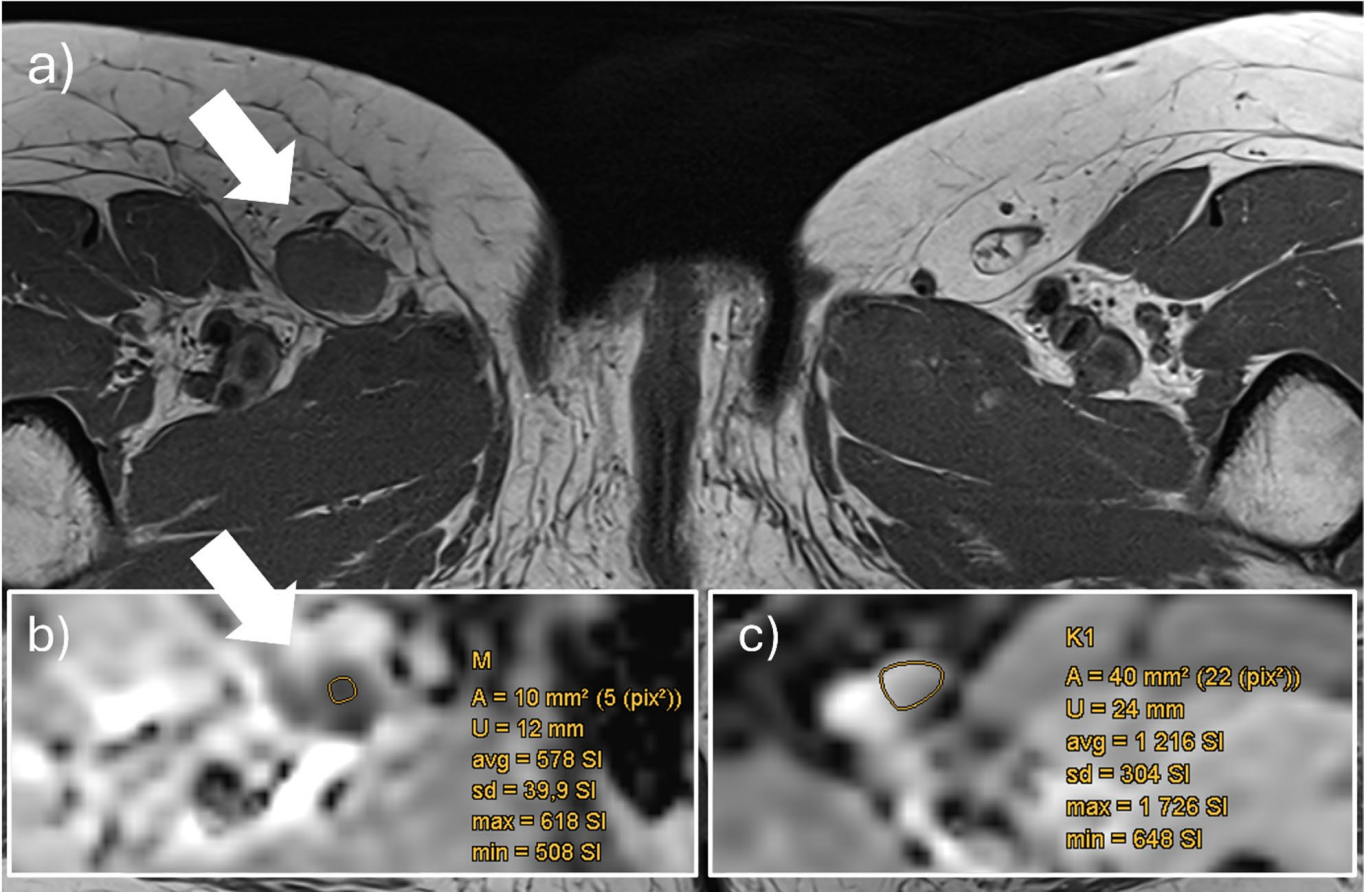
Study patient of MLN group with malignant melanoma on the right lower leg. New lymph node metastasis (white arrow) in the right subinguinal region in the T1w **(a)** and the ADC-MAP (**b**). The corresponding cADC of the metastasis was 0.51 and mADC 0.38 in EPI (cut-off 0.83 and 0.49. *The lower the ratio*,* the higher the probability of a malignant lesion*). One of the three contralateral lymph nodes, measured to calculate cADC, is shown in **c)**. *cADC* Ratio of LN ADC to mean ADC of 3 contralateral LNs, *EPI* Single shot spinecho echoplanar imaging, *mADC* Ratio of LN ADC to ADC of ipsilateral muscle

Optimal EPI cut-offs were 0.83 for cADC (AUC 0.98, 95%-CI 0.96–1, sensitivity 0.99, specificity 0.9) and 0.49 for mADC (AUC 0.99, 95%-CI 0.98–1, sensitivity 0.95, specificity 0.97, Table 4; Fig. 5c). An image example is given in Fig. 6.

For the second reader as well, all metrics were significantly lower in MLNs (see suppl. Table 5).

**Table 4:** Performance of ROC curve analysis within the optimized cut-off of each variable in MLN set

Variable	Cut-off	AUC		CI_l_	CI_u_	Acc	Sens	Spec	PPV	NPV	*R* ^2^
ADC (EPI) (10⁻⁶ mm²/s)	781	0.99*		0.98*	1**	0.95*	0.99*	0.91*	0.93*	0.98*	0.83
ADC (RES) (10⁻⁶ mm²/s)	809	0.83		0.76	0.9*	0.76	0.65	0.88	0.86	0.7	0.23
ADC (VOL) (10⁻⁶ mm²/s)	850	0.97*		0.94*	0.99*	0.9*	0.85	0.94*	0.94*	0.86	0.66
cADC (EPI)	0.83	0.98*		0.96*	1**	0.94*	0.99	0.9*	0.91*	0.98*	0.74
cADC (RES)	0.8	0.83		0.77	0.9*	0.76	0.63	0.91*	0.89	0.69	0.26
cADC (VOL)	0.84	0.91*		0.86	0.96*	0.85	0.88	0.81	0.84	0.86	0.44
mADC (EPI)	0.49	0.99*		0.98*	1**	0.96*	0.95*	0.97*	0.97*	0.94	0.82
mADC (RES)	0.51	0.85		0.78	0.91*	0.79	0.72	0.87	0.86	0.74	0.27
mADC (VOL)	0.57	0.96*		0.93*	0.98*	0.88	0.84	0.93*	0.93*	0.84	0.60
SAD (mm)	7	0.86		0.8	0.92*	0.76	0.75	0.78	0.79	0.74	0.34
Node-RADS	3	0.89		0.84	0.94*	0.81	0.87	0.74	0.78	0.84	0.42

*value > 0.9, **value = 1

*Acc* Accuracy, *ADC* Apparent diffusion coefficients, *AUC* Area under the curve, *CI*_*l*_ Lower confidence interval, *CI*_*u*_ Upper confidence interval, *cADC* Ratio of LN ADC to mean ADC of 3 contralateral LNs, *EPI* Single shot spinecho echoplanar imaging, *mADC* Ratio of LN ADC to ADC of ipsilateral muscle, *MLN* Confirmed metastasized lymph node, *Node-RADS* Node Reporting and Data System, *NPV* Negative predictive value, *PPV* Positive predictive value, *RES* Readout segmentation of long variable echo trains sequence (RESOLVE), *R*^*2*^ McFadden Index, *SAD* Short-axis diameter, *Sens* Sensitivity, *Spec* Specificity, *VOL* Volumetry measuring

### Model validation and agreement with LN status (Gwet´s AC1 Coefficient)

Gradient Boosting (XGBoost) performed best across most ADC parameters. Logistic regression and Neural Networks showed similar performance, followed by Naive Bayes, while KNN and Random Forest performed poorly.

**Fig. 7:**
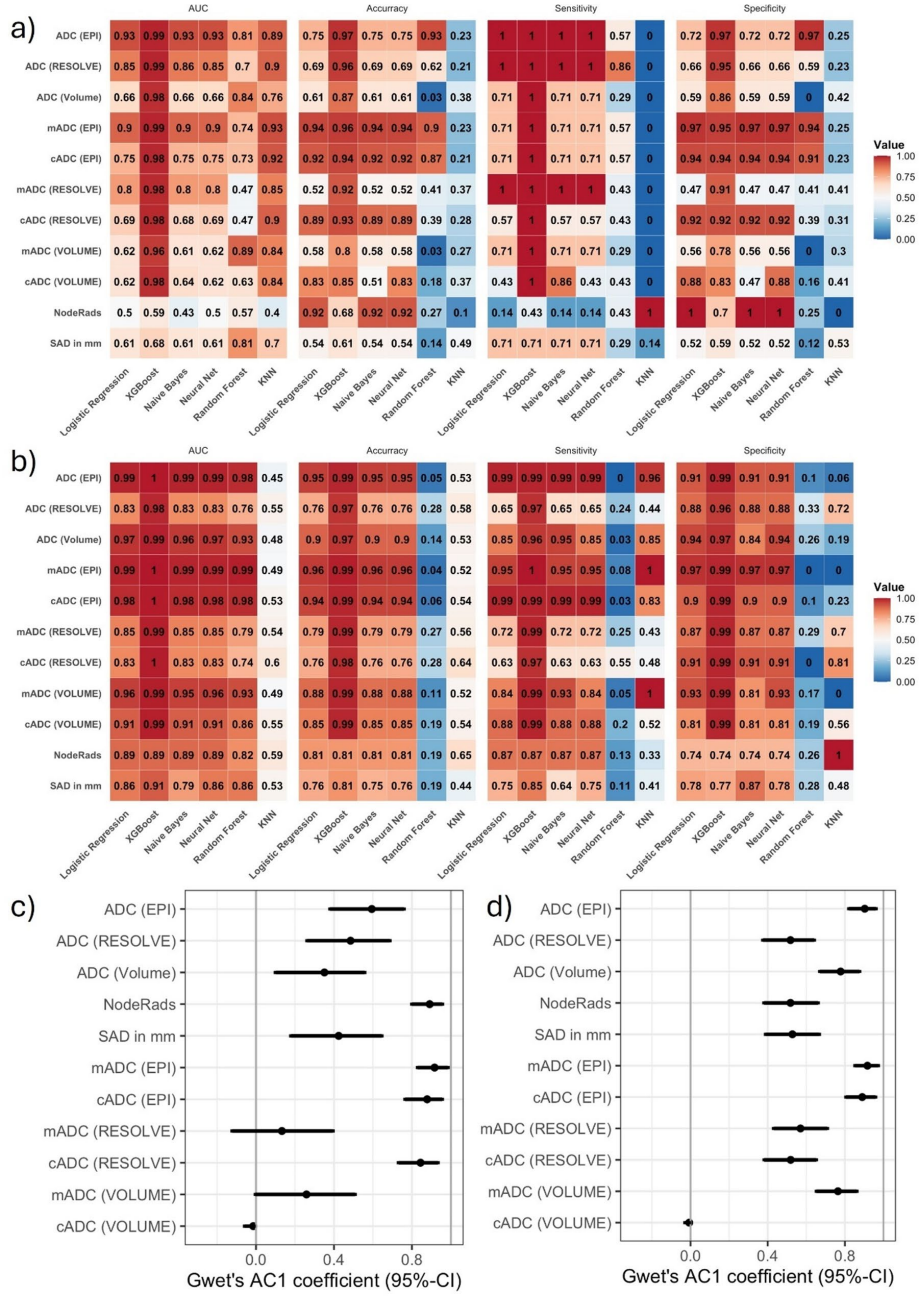
Model validation using machine learning algorithms. Heatmap of performance measures and classifiers for SLN set (**a**) and MLN set (**b**). Gwet’s AC1 agreement with LN status based on logistic-regression–derived optimal cut-offs in the SLN set. (**c**) and MLN set (**d**)

In SLN patients, ADC (EPI) achieved the highest AUC, followed by mADC (EPI) and ADC (RESOLVE). ADC (EPI), ADC (RESOLVE), and mADC (RE-SOLVE) had the highest sensitivity, while mADC (EPI), cADC (EPI), and cADC (RESOLVE) showed the highest specificity and accuracy (Fig. 7a, suppl. Table 6).

In the MLN set, ADC, mADC, and cADC in EPI showed the best performance (Fig. 7b, suppl. Table 7). Agreement with LN status was highest for mADC (EPI) in both SLN (0.92, 95%-CI 0.83–0.98) and MLN set (0.92, 95%-CI 0.85–0.97), followed by Node-RADS in the SLN set and cADC (EPI) in both sets (Fig. 7c/d, suppl. Table 8).

Across the six evaluated models, performance differences were generally small: XGBoost achieved the highest values, logistic regression and Neural Networks performed similarly well, and Naive Bayes followed closely. Only KNN and Random Forest showed noticeably lower performance, which is consistent with their known limitations in small, low-dimensional datasets.

### Inter-reader agreement and cut-off validation

The ADC metrics in EPI demonstrated good to excellent inter-reader agreement with only a small bias (see Tabl. 5). The highest ICC among all ADC metrics was observed for ADC (EPI) at 0.89 (95% CI: 0.85–0.92), with a bias of 2.5 × 10⁻⁶ mm²/s and limits of agreement (LoA) from − 207 to 212 × 10⁻⁶ mm²/s. This was followed by cADC (EPI) (ICC = 0.87 [95% CI: 0.82–0.90], bias = − 0.02, LoA = − 0.26 to 0.22) and mADC (EPI) (ICC = 0.84 [95% CI: 0.78–0.88], bias = − 0.006, LoA = − 0.18 to 0.17).

Among the morphological parameters, inter-reader agreement for SAD was excellent, with an ICC of 0.94 (95% CI: 0.92–0.96), a bias of − 0.42 mm, and limits of agreement (LoA) from − 4.67 to 3.83 mm, while Node-RADS showed moderate agreement with an ICC of 0.71 (95% CI: 0.57–0.80), a bias of − 0.40, and LoA from − 2.29 to 1.50.

**Table 5:** ADC metrics and morphological parameters assessed by both readers for all positive and negative LNs, and corresponding inter-reader agreement

Variable	All LNs (*n* = 151)	All positive LNs (*n* = 82)	All negative LNs (*n* = 69)	*p*-value *p* vs. *n*	ICC 2,1	Bias	LoA (95% KI)
ADC (EPI) R1	732 (616, 922)	624,5 (569.5, 787.5)	943 (863,1077)	< 0.001			
ADC (EPI) R2	760 (616,907)	642 (560, 730.25)	914 (834,1038.5)	< 0.001	0.89 (0.85, 0.92)	2,5	-207, 212
ADC (RES) R1	855 (746, 1027)	763 (693.25, 874.5)	990 (856.5,1114.5)	< 0.001			
ADC (RES) R2	846 (706, 1041)	731 (652,866.75)	964 (841, 1134,5)	< 0.001	0.80 (0.74, 0.85)	12.3	-257, 281
mADC (EPI) R1	0.48 (0.41, 0.61)	0.41 (0.37, 0.46)	0.62 (0.56, 0.73)	< 0.001			
mADC (EPI) R2	0.51 (0.42, 0.60)	0.44 (0.38, 0.50)	0.60 (0.54, 0.69)	< 0.001	0.839 (0.78, 0.88)	-0.006	-0.18, 0.17
mADC (RES) R1	0.52 (0.45, 0.61)	0.46 (0.40, 0.53)	0.61 (0.52, 0.68)	< 0.001			
mADC (RES) R2	0.57 (0.47, 0.69)	0.52 (0.44, 0.59)	0.62 (0.55, 0.73)	< 0.001	0.70 (0.49, 0.81)	-0.06	-0.26, 0.15
cADC (EPI) R1	0.78 (0.63, 1.01)	0.64 (0.57, 0.76)	1.01 (0.91, 1.08)	< 0.001			
cADC (EPI) R2	0.81 (0.65, 0.99)	0.69 (0.58, 0.81)	1.02 (0.87, 1.15)	< 0.001	0.87 (0.82, 0.90)	-0.02	-0.26, 0.22
cADC (RES) R1	0.85 (0.67, 0.99)	0.71 (0.55, 0.87)	0.96 (0.85, 1.10)	< 0.001			
cADC (RES) R2	0.88 (0.67, 1.04)	0.74 (0.55, 0.95)	0.99 (0.84, 1.11)	< 0.001	0.72 (0.63, 0.79)	-0.05	-0.44, 0.35
SAD R1	7.00 (5.00, 11.00)	9 (7, 15)	5 (4, 7)	< 0.001			
SAD R2	7.00 (6.00, 10.00)	10 (7, 17)	6 (4, 7)	< 0.001	0.941 (0.92, 0.96)	-0,42	-4.67, 3.83
NodeRADS R1	3.00 (2.00, 4.00)	3.5 (3, 5)	2 (1, 3)	< 0.001			
NodeRADS R2	3.00 (2.00, 4.00)	4 (3, 5)	2 (2,3)	< 0.001	0.708 (0.57, 0.80)	-0.40	-2.29, 1.50
ROI (EPI) R1	8 (5,13)	7 (4,12)	10 (6,14)	0.015			
ROI (EPI) R2	7 (4,10)	6.5 (4,10)	7 (4.5,10)	0.706	0,68 (0.58, 0.76)	1.23	-7,7,10
ROI (RES) R1	9 (6, 14)	10 (5.75, 16)	9 (6,11.5)	0.536			
ROI (RES) R2	8 (5,11)	8 (5, 12.25)	8 (5.5, 10.5)	0.759	0.71 (0.59, 0.74)	1.82	-7.9, 11.5

*ADC* Apparent diffusion coefficient (in 10⁻⁶ mm²/s), *cADC* Ratio of LN ADC to mean ADC of 3 contralateral LNs, *EPI* Single shot spinecho echoplanar imaging, *mADC* Ratio of LN ADC to ADC of ipsilateral muscle, *LN* lymph node, *Node-RADS* Node Reporting and Data System, *R1* Reader 1, *R2* Reader 2 *RES* Readout segmentation of long variable echo trains sequence, *SAD* Short-axis diameter (in mm), *ROI* Region of interest

There was no significant correlation between the ROI size and ADC (EPI) for both readers (R1: Spearman’s ρ = 0.05, *p* = 0.538; R2: ρ = − 0.042, *p* = 0.61).

In contrast, a significant but weak positive correlation was observed between ROI size and ADC (RES) (R1: ρ = 0.337, *p* < 0.01; R2: ρ = 0.266, *p* < 0.01).

### Inter-reader cut-off validation

When validating the previously derived SLN cut-off (Reader 1) on the full LN dataset of Reader 2, mADC (EPI) and cADC (RES) showed the best performance (Table 6), both yielding the highest Youden Index (Y) of 0.84 (mADC (EPI): sensitivity 0.98, specificity 0.86; cADC (RES): sensitivity 1, specificity 0.84). These were closely followed by cADC (EPI) (Y 0.83, sensitivity 1, specificity 0.83), whereas the absolute ADC values showed lower discriminatory performance (Table 6).

**Table 6:** Validation of the cut-off values derived from the SLN set (Reader 1) on all LNs measured by reader 2

ADC (EPI)	TP	TN	FP	FN	Sens	Spec	PPV	NPV	Acc	Y
ADC (EPI)	81	48	21	1	0.99	0.7	0.79	0.98	0.85	0.68
ADC (RES)	67	42	27	15	0.82	0.61	0.71	0.74	0.72	0.43
mADC (EPI)	57	59	10	1	0.98	0.86	0.85	0.98	0.91	0.84
mADC (RES)	65	41	28	0	1	0.59	0.7	1	0.79	0.59
cADC (EPI)	62	57	12	0	1	0.83	0.84	1	0.91	0.83
cADC (RES)	44	58	11	0	1	0.84	0.8	1	0.9	0.84
SAD	78	32	37	4	0.95	0.46	0.68	0.89	0.73	0.41
Node-RADS	34	69	0	48	0.41	1	1	0.59	0.68	0.41

*ADC* Apparent diffusion coefficient, *cADC* Ratio of LN ADC to mean ADC of 3 contralateral LNs, *EPI* Single shot spinecho echoplanar imaging, *mADC* Ratio of LN ADC to ADC of ipsilateral muscle, *LN* lymph node, *Node-RADS* Node Reporting and Data System, *RES* Readout segmentation of long variable echo trains sequence, *SAD* Short-axis diameter

### Inter-set cut-off validation

When validating the previously derived SLN cut-off on the MLN dataset (Reader 1), mADC (EPI) showed the best performance with a Y of 0.92 (sensitivity 0.95, specificity 0.97), followed by cADC (EPI) with a Y of 0.86 (sensitivity 0.93, specificity 0.93), with a clear margin over all other parameters (Table 7).

**Table 7:** Validation of the cut-off values derived from the SLN set (Reader 1) on MLN set (Reader 1)

ADC (EPI)	TP	TN	FP	FN	Sens	Spec	PPV	NPV	Acc	Y
ADC (EPI)	75	50	19	0	1	0.72	0.8	1	0.87	0.72
ADC (RES)	62	47	22	13	0.83	0.68	0.74	0.78	0.76	0.51
ADC (VOL)	75	41	28	0	1	0.59	0.73	1	0.81	0.59
cADC (EPI)	70	64	5	5	0.93	0.93	0.93	0.93	0.93	0.86
cADC (RES)	43	64	5	32	0.57	0.93	0.9	0.67	0.74	0.5
cADC (VOL)	61	60	9	14	0.81	0.87	0.87	0.81	0.84	0.68
mADC (EPI)	71	67	2	4	0.95	0.97	0.97	0.94	0.96	0.92
mADC (RES)	67	35	34	8	0.89	0.51	0.66	0.81	0.71	0.4
mADC (VOL)	75	40	29	0	1	0.58	0.72	1	0.8	0.58
SAD	74	36	33	1	0.99	0.52	0.69	0.97	0.76	0.51
Node-RADS	25	69	0	50	0.33	1	1	0.58	0.65	0.33

*ADC* Apparent diffusion coefficient (in 10⁻⁶ mm²/s), *cADC* Ratio of LN ADC to mean ADC of 3 contralateral LNs, *EPI* Single shot spinecho echoplanar imaging, *mADC* Ratio of LN ADC to ADC of ipsilateral muscle, *LN* lymph node, *Node-RADS* Node Reporting and Data System, *RES* Readout segmentation of long variable echo trains sequence, *SAD* Short-axis diameter

## Discussion

In this prospective study, we evaluated the diagnostic performance of a standardized ADC ratio cut-off for identifying lymph node (LN) metastasis in early and advanced malignant melanoma (MM), addressing variability and dependences of absolute ADC values.

### Main findings

In the advanced-stage group (MLN set), malignant lymph nodes were most accurately identified using the cADC ratio in echo-planar imaging (EPI), defined as the quotient of the target node ADC and the ADC of the patient’s benign contralateral nodes. This parameter achieved an AUC of 0.98 (sensitivity 0.99, specificity 0.90) and clearly outperformed Node-RADS (AUC 0.89) and SAD (AUC 0.86).

The optimal cADC cut-off was 0.83, indicating malignancy when the ADC of a node was < 83% of the contralateral benign reference. Similarly, the mADC ratio, comparing target-node ADC to adjacent muscle tissue, showed excellent diagnostic performance (AUC 0.99; sensitivity 0.95, specificity 0.97; agreement 0.92) with an optimal cut-off of 0.49.

In the early-stage SLN set, metastatic nodes likewise exhibited significantly lower ADC and ratio values (cADC and mADC) in EPI sequence, while performance of both Node-RADS and SAD remained limited and did not significantly differentiate metastatic from benign SLNs. Ratio-based diagnostics remained moderate to good in this group despite the typically smaller node size.

Across both patient sets, optimal cut-offs for the ratios in EPI were highly consistent: 0.81 for cADC in the SLN set and 0.83 in the MLN set, and 0.49 for mADC in both sets. Similar thresholds in different sequences (EPI and RESOLVE) and measurement techniques (ROI and VOI) highlight the consistency and practical value of these ADC ratios. Analysis of the agreement with LN status (Gwet´s AC1 Coefficient) showed that mADC (EPI) was most consistent overall, followed by absolute ADC (EPI) and cADC (EPI). Across both patient sets, optimal cut-offs for the ratios in EPI were highly consistent: 0.81 for cADC in the SLN set and 0.83 in the MLN set, and 0.49 for mADC in both sets. Similar thresholds in different sequences (EPI and RESOLVE) and measurement techniques (ROI and VOI) highlight the consistency and practical value of these ADC ratios. Analysis of the agreement with LN status (Gwet´s AC1 Coefficient) showed that mADC (EPI) was most consistent overall, followed by absolute ADC (EPI) and cADC (EPI). In the cut-off validation using the independent measurements of a blinded second reader, the EPI-based ratios demonstrated the best performance. Additionally, the ADC metrics in EPI showed a high inter-reader agreement (ADC: ICC 0.89 (95% CI: 0.85–0.92, cADC: 0.87 [95% CI: 0.82–0.90] and mADC: 0.84 [95% CI: 0.78–0.88]). These values are comparable to the reliabilities reported for absolute ADCs by Papoutsaki et al. (2021), evaluating 100 cervical LNs of head and neck squamous cell carcinoma patients (ICC 0.88); Teixeira et al. (2025), analyzing 223 LNs in endometrial carcinoma patients (ICC 0.81 to 0.96); and Noto et al. (2025), evaluating 359 LNs in prostate cancer patients (ICC 0.93) [43–45].

Our findings indicate potential cut-offs that may aid the clinical assessment of LNs in DWI: approximately a 17–19% ADC reduction relative to contralateral nodes (cADC 0.81–0.83) and a 51% reduction relative to adjacent muscle (mADC 0.49). These internally normalized metrics demonstrate high diagnostic accuracy and consistency across disease stages, supporting their potential utility in clinical LN evaluation.

### Robustness of ADC-based findings across ML modelling frameworks

Five commonly used machine-learning algorithms representing different model families (tree-based, boosting, distance-based, probabilistic, and neural approaches) were included to verify that the diagnostic performance patterns and cut-off estimates derived from logistic regression were robust across diverse modelling assumptions. As expected for small, low-dimensional biomedical datasets, Gradient Boosting (XGBoost) performed best, which is consistent with its known ability to capture nonlinear relationships and handle complex interactions efficiently. Neural Networks and logistic regression yielded similar results due to the simple model structure and limited feature space. Random Forest and KNN showed lower performance, potentially due to the small dataset where these methods are more sensitive to noise or local variability. Naive Bayes performed moderately well, consistent with its probabilistic nature and robustness in low-dimensional settings. Notably, XGBoost occasionally generated implausible cut-offs (e.g., cADC > 1), suggesting that such isolated outputs are more likely attributable to overfitting in a small, low-dimensional dataset than to a biologically meaningful phenomenon.

### Clinical interpretation in light of previous studies

To our knowledge, this study is among the first to specifically evaluate ADC values, their intra-individual tissue ratios, and corresponding diagnostic cut-offs in both early and advanced stages of MM. One likely reason for the scarcity of prior data is that MRI has not yet been integrated into routine LN staging for MM [1, 4, 46], unlike in breast or prostate cancer, thereby limiting its availability for retrospective analyses.

Knill et al. (2023) reported absolute ADC values with a median of 1420 mm²/s (10th – 90th percentile: 810–2260 mm²/s) for malignant LNs in advanced-stage MM patients [21]. These values are substantially higher than those observed in our study, likely reflecting differences in scanner hardware and sequence parameters and highlighting the limited comparability and clinical applicability of absolute ADC values across studies [7, 24–26].

In early-stage MM patients, Schaarschmidt et al. (2018) found no significant differences in absolute ADC values between positive and negative SLNs [12], which may be attributed to a voxel volume approximately six times larger than in our study, potentially compromising diagnostic resolution.

Based on previously published studies on absolute ADC values in other tumor entities, retrospectively calculated proportions of malignant and benign LNs for each study would fall below our cADC cut-offs [19, 23, 47–49].

However, these proportions were calculated across all patients, while our ratios compare the same patient’s LNs, which is easily applicable in daily routine. By comparing LNs within the same patient, our method provides internal referencing and better standardization suitable for clinical use, intending to overcome variability and dependences of absolute ADC values.

### Diagnostic implications for sentinel and non-sentinel lymph nodes

Beyond comparisons with existing literature, our findings have important implications for clinical decision-making. Considering clinical application, both ratios appear to be very specific, reliable and accurate in SLN-settings, potentially avoiding unnecessary following therapeutic interventions. Due to moderate sensitivity (0.71), especially given the low positivity rate of SLNE (0.1), neither cADC nor mADC ratios from DWI can currently replace sentinel lymph node extraction (SLNE). Nevertheless, in the MLN set with still predominantly subcentimeter metastases, cADC and mADC demonstrated a notable diagnostic advantage over conventional morphological criteria like LN size and configuration. As functional, quantitative, normalized, and widely accessible markers, ADC ratios may serve as an effective tool in clinical surveillance to monitor at-risk LN regions and confirm the diagnosis of locoregional recurrence. Few, if any, studies have specifically examined Node-RADS in malignant melanoma. In our MLN set, the optimal Node-RADS cut-off was ≥ 3, which aligns with recommendations from the original publication [9] and showing comparable performance to pooled results (AUC 0.92) from a meta-analysis of other tumour entities [11]. However, Node-RADS was not suitable for distinguishing initially metastasized from benign SLNs (*p* = 0.98), likely due to the typically low tumor burden at this stage, which may be insufficient to cause detectable changes in LN size or configuration. Additionally, Node-RADS showed only a moderate interreader agreement (0.708, 95% CI 0.57, 0.80), suggesting some subjective bias, which is aligned with observation of a meta-analysis (2025) in other tumor entities [11]. Similarly, the more labor-intensive VOI-based ADC-analysis did not provide additional benefits.

### Technical considerations and potential confounders

In addition to the clinical relevance, technical factors must be considered when integrating ADC measurements into routine practice. Introducing ADC metrics into routine MM surveillance requires consideration of sequence parameters and potential confounders. Our acquisition protocol included two b-values: *b = 50* s/mm² (low) and *b = 800* s/mm² (high). While the acquisition with more than two b-values can improve the accuracy of ADC estimation [50], limiting the sequence to two values reduces acquisition time, making the sequence less susceptible to motion artifacts, and improves feasibility in cancer patients [50–52], while maintaining comparable diagnostic performance to protocols using multiple b-values [51, 53].

It is known that *b = 50* generally does not fully suppress perfusion effects [54]. However, LNs seem to be less affected by this limitation, possibly due to their insulation within poorly vascularized adipose tissue [51]. As a result, even *b = 0* seems to be diagnostically usable in this context [19, 23, 26, 48, 55].

A previous study on b-value optimization for detection MM metastasis recommended *b = 50* (low) and 750–1100 s/mm² (high) to balance SNR and distortion [21]. The selection of *b = 800* in our study aligns with these recommendations, ensures distance from the noise floor in higher b-values [24], and is supported by recommendations for 3T-MRI-protocols in MM and other tumor entities [50], as well as prior studies evaluating mediastinal LNs [51] and axillary LNs in breast cancer [19].

Although no confounding factors like LN inflammation or alterations in muscle were observed in our histological or follow-up results, they potentially could influence normalization. Previous studies suggested that inflamed LNs usually exhibit higher ADC values than malignant LNs [55–57]. Consequently, inflammation in the target or reference LN is more likely to yield a true-negative cADC or mADC-ratio, which is diagnostically valuable in cancer patients.

In contrast, an undetected malignancy in reference LNs could potentially mask a malignant target LN in cADC-ratio. However, mADC would remain unaffected, and averaging three contralateral LNs would minimize this risk. Conversely, in patients with in T1/2w visible alterations in muscle like sarcopenia, edema, or fibrosis, cADC should be prioritized. Beyond that, muscle tissue is anatomically accessible in most nodal regions and is among the most homogeneous tissues, making it a common reference in quantitative MRI-sequence comparisons [58].

Alternative approaches in breast cancer patients proposed the corresponding primary tumors as the reference tissue for LN ratios [22, 59]. However, this is not feasible in MM because of the typically thin and usually already excised primary lesion.

### Limitations

First, due to the comparatively lower-than-expected positive rate in SLNE and the monocentric design, we observed a limited number of positive SLNs [60, 61]. To address this, we included a second patient group (MLN) with more positive LNs for cut-off verifications, further analysis, and enhanced statistical power. We also validated our findings using machine-learning classifiers, three of which (including logistic regression) confirming our proposed thresholds.

Second, prior to data acquisition, the applied sequences were visually evaluated but not tested in a separate phantom study. Such validation could have provided a benchmark for assessing technical confounders and are crucial in multicenter studies that combine absolute ADC values across different scanners and sequences [54]. In contrast, we used relative ADC values, the same scanner and a standardized sequence protocol throughout the study, including uniform patient positioning, coil placement, and padding.

Third, our monocentric design limits generalizability. External validation of the proposed ratio cut-offs across different institutions, scanners, protocols, and tumor types would strengthen their applicability, particularly in larger cohorts of patients with positive SLNs.

### Perspectives

Finally, the performance of ADC-ratios in the SLN set was only impacted by two outlier LNs containing micrometastases (1 und 1.5 mm, respectively). Detecting micrometastases is clinically relevant, as melanoma-specific survival already worsens when metastases reach or exceed 0.3 mm (80.3% vs. 94.1%) [60]. In current clinical practice, even small LN metastases result in upstaging to at least AJCC stage IIIa, necessitating additional treatments [1, 4, 46]. While completion LN dissection was formerly standard for micrometastatic SLNs, it is now largely omitted [3, 46], as no survival benefit has been demonstrated over active surveillance [62]. Weighing the benefits and potential risks of immune checkpoint inhibitors or targeted therapies, current European and American guidelines (2025) discuss a LN metastasis size of ≥ 1 mm as a possible threshold for initiating adjuvant systemic therapies, although they are formally approved from stage IIIa onwards [3, 46]. In summary, future imaging modalities should aim to reliably detect LN metastases of approximately 1 mm in diameter. Achieving this remains a technical challenge, as MRI is constrained by spatial resolution, while PET is limited by the metabolic activity of metastases, with lesions showing low tracer uptake potentially remaining below the detection threshold [12, 63]. Future studies might also explore automated software-assisted ADC-evaluation of LNs, including direct cADC and mADC calculation.

## Conclusion

Relative ADC-ratios offer non-invasive method for differentiating benign from metastatic lymph nodes, providing improved reproducibility and standardization through internal referencing. Whilst SLNE remains the diagnostic gold standard, DWI-MRI could serve as an important complementary diagnostic tool to assess patients with clinically suspicious lymph nodes.

## Supplementary Information

Below is the link to the electronic supplementary material.

Supplementary Material 1

## Data Availability

The datasets used and/or analysed during the current study are available from the corresponding author on reasonable request.

## Abbreviations

Acc: Accuracy
ADC: Apparent diffusion coefficient in × 10⁻⁶ mm²/s
AJCC: American joint committee on cancer
AUC: Area under the curve
CIl: Lower confidence interval
cLN: contralateral lymph node
CIu: Upper confidence interval
cADC: Ratio of LN ADC to mean ADC of 3 contralateral LNs
CU: Cut-off
CUP: Cancer of unknown primary localisation
EPI: Single shot spinecho echoplanar imaging
EST: Estimation
KNN: K-Nearest neighbors
mADC: Ratio of LN ADC to ADC of ipsilateral muscle tissue
MLN: Confirmed metastasized lymph node
MM: Malignant melanoma
Node-RADS: Node reporting and data system
NPV: Negative predictive value
PE: Primary excision
PPV: Positive predictive value
R^2^: McFadden index
RES: Readout segmentation of long variable echo trains sequence (RESOLVE)
SAD: Short-axis diameter
SAR: Specific absorption rate
Sens: Sensitivity
SLNE: Sentinel lymph node extraction
Spec: Specificity
SPECT: Single photon emission computed tomography
SSM: Superficial spreading melanoma
tLN: Target lymph node
VOL: Volumetry measuring
XGBoost: Gradient boosting
