# Supplementary material for: Differentiating lymph node status in malignant melanoma: the role of apparent diffusion coefficient ratio in diffusion-weighted MRI – a prospective diagnostic study
Source: BMC Med Imaging. 2026 Feb 4;26:108. doi: 10.1186/s12880-026-02205-6 (PMC12930938; doi:10.1186/s12880-026-02205-6)
Supplement: Supplementary file 1 — Supplementary Material 1 [file 12880_2026_2205_MOESM1_ESM.docx]

# Supplementary Material

**Supplementary Tab. 1** MRI sequence parameters

| **Parameters** | **EPI** | **RESOLVE** | **T1** | **T2 Dixon** |
| --- | --- | --- | --- | --- |
| FOV (mm^2^) | 350 x 350 | 350 x 350 | 350 x 350 | 350 x 350 |
| Matrix | 128 x 128 | 128 x 128 | 448 x 358 | 320 x 256 |
| Slices | 50 | 50 | 50 | 50 |
| Slice thickness (mm) | 3 | 3 | 3 | 3 |
| Gap (mm) | 0 | 0 | 0 | 0 |
| Voxel size (mm^3^) | 1,4 x 1,4 x 3 | 1,4 x 1,4 x 3 | 0,4 x 0,4 x 3 | 0,5 x 0,5 x 3 |
| Orientation | transversal | transversal | transversal | transversal |
| Scan duration (minutes) | 6:39 | 07:07 | 3:04 | 2:35 |
| B-values | 50, 800 | 50, 800 | - | - |
| Diffusion mode | 4-Scantrace | 4-Scantrace | - | - |
| Directions | 4 | 4 | - | - |
| Averages | 2, 10 | 1, 3 | 1 | 1 |
| Fat saturation | spectral, strong | spectral, strong | standard | Dixon |
| Repetiton time (ms) | 7800 | 4130 | 552 | 3650 |
| Echo time (ms) | 62 | 53, 88 | 17 | 78 |
| Acceleration mode | GRAPPA | SMS, 7 segments | GRAPPA | GRAPPA |
| Acceleration factor | 2 | 2 | 3 | 3 |
| Bandwith (Hz/Px) | 2170 | 930 | 349 | 558 |
| Echo spacing (ms) | 0,54 | 0,30 | 8,50 | 9,69 |

*EPI* single-shot spinecho echoplanar imaging, *RESOLVE* readout segmentation of long variable echo-trains sequence

**Supplementary Tab. 2** Aditional patients and tumour characteristics of SLN-set. In brackets interquartile range (IQR) or percentages (%).

| **Variable** | **Overall** | **Positive** | **Negative** | **p-value** |
| --- | --- | --- | --- | --- |
| Patients | **52** | **7** | **45** |  |
| Age (y; median, IQR) | 66.50 (60.5, 75.5) | 66 (60.5, 80) | 67 (61, 75) | 0.788 |
| Male (n; %) | 26 (50.0) | 4 (57.1) | 22 (48.9) | 1.00 |
| Female (n; %) | 26 (50.0) | 3 (42.9) | 23 (51.1) |  |
| **MM characteristics** |  |  |  |  |
| Nodular (n; %) | 22 (42.3) | 6 (85.7) | 16 (35.6) | 0.043 |
| SSM (n; %) | 26 (50) | 1 (14.3) | 25 (55.6) |  |
| Others (n; %) | 4 (7.7) | 0 | 4 (8.88) |  |
| Depth diameter  (mm; median, IQR) | 1.85 (1.3, 3.35) | 3.2 (2.74, 5.75) | 1.7 (1.2, 2.4) | 0.011 |
| Ulceration (n; %) | 24 (46.2) | 5 (71.4) | 19 (42.2) | 0.301 |
| **MM localisations** |  |  |  | 0.163 |
| Arm/shoulder (n; %) | 15 (28.8) | 0 ( 0.0) | 15 (33.3) |  |
| Leg (n; %) | 18 (34.6) | 2 (28.6) | 16 (35.6) |  |
| Head/neck (n; %) | 4 ( 7.7) | 1 (14.3) | 3 ( 6.7) |  |
| Trunk (n; %) | 15 (28.8) | 4 (57.1) | 11 (24.4) |  |
| Days between PE-MRI  (d; median, IQR) | 32 (26, 35.25) | 28 (20.5, 36) | 32 (26, 35) | 0.493 |
| Days between MRI-SLNE (d; median, IQR) | 0 (0, 3) | 0 (0, 2) | 0 (0, 3) | 0.933 |
| **AJCC stage** |  |  |  |  |
| Ib (n; %) | 23 (44.2) | 0 ( 0.0) | 23 (51.1) | 0.137 |
| IIa (n; %) | 12 (23.1) | 3 (42.9) | 9 (20.0) |  |
| IIb (n; %) | 6 (11.5) | 1 (14.3) | 5 (11.1) |  |
| IIc (n; %) | 9 (17.3) | 3 (42.9) | 6 (13.3) |  |
| III (n; %) | 1 ( 1.9) | 0 ( 0.0) | 1 ( 2.2) |  |
| IIIc (n; %) | 1 ( 1.9) | 0 ( 0.0) | 1 ( 2.2) |  |

AJCC American Joint Committee on Cancer, MM Malignant melanoma, PE Primary excision of cutanous melanoma, SLNE Sentinel lymph node extraction, SSM Superficial spreading melanoma.

**Supplementary Tab. 3** Additional patients and tumour characteristics of MLN-set. In brackets interquartile range (IQR) or percentages (%).

| **Variable** | **Overall** | **MLN group** | **Negative group*** | **p-value** |
| --- | --- | --- | --- | --- |
| **Patients** | **60** | **12** | **48** |  |
| Age (y; median, IQR) | 68.5 (61,77.5) | 73 (66.25, 77.50) | 67 (59.5, 76.25) | 0.426 |
| Male (n; %) | 31 (52) | 8 (66.7) | 23 (48) | 0.401 |
| Female (n; %) | 29 (48.3) | 4 (33.3) | 25 (52.1) |  |
| **MM characteristics** |  |  |  |  |
| CUP (n; %) | 3 ( 5.0) | 3 (25.0) | 0 (0) | <0.001 |
| Nodular (n; %) | 24 (40.0) | 5 (41.7) | 19 (39.6) |  |
| SSM (n; %) | 26 (43.3) | 1 (8.3) | 25 (52.1) |  |
| Others (n; %) | 7 (11.7) | 3 (25.0) | 4 ( 8.3) |  |
| Depth diameter  (mm; median, IQR) | 1,8 (1.3, 3.1) | 2.20 (1.61, 2.70) | 1,7 (1.28, 3.12) | 0.562 |
| Ulceration (n; %) | 28 (50) | 7 (87.5) | 21 (43.8) | 0.056 |
| **MM localisation** |  |  |  |  |
| Arm/shoulder (n; %) | 17 (28.3) | 2 (16.7) | 15 (31.2) | 0.007 |
| Trunk (n; %) | 16 (26.7) | 4 (33.3) | 12 (25) |  |
| Legs (n; %) | 19 (31.7) | 2 (16.7) | 17 (35.4) |  |
| CUP (n; %) | 3 (5.0) | 3 (25) | 0 (0) |  |
| Head/neck (n; %) | 5 ( 8.3) | 1 (8.3) | 4 ( 8.3) |  |
| **AJCC stage** |  |  |  |  |
| Ib (n; %) | 23 (38.3) | 0 (0) | 23 (47.9) | <0.001 |
| IIa (n; %) | 10 (16.7) | 0 (0) | 10 (20.8) |  |
| IIb (n; %) | 6 (10.0) | 0 (0) | 6 (12.5) |  |
| IIc (n; %) | 7 (11.7) | 0 (0) | 7 (14.6) |  |
| III (n; %) | 1 ( 1.7) | 0 (0) | 1 (2.1) |  |
| IIIb-c (n; %) | 1 ( 1.7) | 1 (8.3) | 0 (0) |  |
| IIIc (n; %) | 1 ( 1.7) | 0 (0) | 1 (2.1) |  |
| IV (n; %) | 10 (16.7) | 10 (83.3) | 0 (0) |  |

*SLNE group with all negative lymph nodes.

AJCC American Joint Committee on Cancer, CUP Cancer of unknown primary localisation, MM Malignant melanoma, MLN Confirmed metastasized lymph node, PE Primary excision, SLNE Sentinel lymph node extraction, SSM Superficial spreading melanoma.

**Supplementary Tab. 4** SLN set Reader 2

| **Variable** | **all** | **Positive SLNs** | **Negative SLNs** | **p-value** |
| --- | --- | --- | --- | --- |
| ADC (EPI) | 898 (794, 1028) | 688 (615, 731) | 912.5 (838.75, 1030.5) | <0,001 |
| ADC (RES) | 956 (840, 1118) | 913 (723, 1041) | 960 (841, 1127.75) | 0.088 |
| SAD | 6 (4, 7) | 6 (4, 9) | 6 (4.25, 7) | 0.899 |
| NR | 2 (2, 3) | 3 (1, 4) | 2 (2, 3) | 0.1 |
| mADC (EPI) | 0.59 (0.52, 0.68) | 0.48 (0.40, 0.50) | 0.61 (0.54, 0.69) | 0.002 |
| mADC (RES) | 0.62 (0.54, 0.71) | 0.64 (0.48, 0.70) | 0.62 (0.55, 0.72) | 0.219 |
| cADC (EPI) | 0.99 (0.83, 1.09) | 0.80 (0.65, 0.93) | 1.02 (0.88, 1.14) | 0.012 |
| cADC (RES) | 0.96 (0.86, 1.11) | 0.95 (0.70, 1.09) | 0.99 (0.84, 1.09) | 0.563 |
| ROI EPI | 5 (7,10) | 4 (5, 6) | 5 (7, 10) | 0.108 |
| ROI RES | 5 (7,10) | 4 (5, 6) | 6 (8, 10) | 0.009 |

ADC Apparent diffusion coefficient (in 10⁻⁶ mm²/s), cADC Ratio of LN ADC to mean ADC of 3 contralateral LNs, EPI Single shot spinecho echoplanar imaging, mADC Ratio of LN ADC to ADC of ipsilateral muscle, LN lymph node, NR Node Reporting and Data System, RES Readout segmentation of long variable echo trains sequence, SAD Short-axis diameter (in mm), *ROI* Region of interest.

**Supplementary Tab. 5** MLN set Reader 2

| **Variable** | **all** | **Positive SLNs** | **Negative SLNs** | **p-value** |
| --- | --- | --- | --- | --- |
| ADC (EPI) | 774.5 (617, 913.25) | 641 (540, 730) | 914 (834, 1038.5) | <0.001 |
| ADC (RES) | 844 (705.25, 1044.25) | 726 (652, 853) | 964 (841, 1134.5) | <0.001 |
| SAD | 8 (6 10) | 8 (10, 18) | 4 (6, 7) | <0.001 |
| NR | 3 (2, 4) | 4 (3, 5) | 2 (2, 3) | <0.001 |
| mADC (EPI) | 0.52 (0.42, 0.61) | 0.44 (0.37, 0.51) | 0.60 (0.54, 0.69) | <0.001 |
| mADC (RES) | 0.57 (0.47, 0.69) | 0.49 (0.44, 0.58) | 0.62 (0.55, 0.73) | <0.001 |
| cADC (EPI) | 0.81 (0.65, 1.01) | 0.67 (0.57, 0.80) | 1.02 (0.87, 1.15) | <0.001 |
| cADC (RES) | 0.87 (0.66, 1.04) | 0.74 (0.53, 0.95) | 0.99 (0.84, 1.11) | <0.001 |
| ROI EPI | 7.00 (4.25, 10.00) | 7.00 (4.00, 10.00) | 7.00 (4.50, 10.00) | 0.949 |
| ROI RES | 8.00 (5.00, 11.75) | 9.00 (5.00, 13.00) | 8.00 (5.50, 10.50) | 0.384 |

ADC Apparent diffusion coefficient (in 10⁻⁶ mm²/s), cADC Ratio of LN ADC to mean ADC of 3 contralateral LNs, EPI Single shot spinecho echoplanar imaging, mADC Ratio of LN ADC to ADC of ipsilateral muscle, LN lymph node, NR Node Reporting and Data System, RES Readout segmentation of long variable echo trains sequence, SAD Short-axis diameter (in mm), *ROI* Region of interest.

**Supplementary Tab. 6 Model validation using machine learning algorithms: performance measures for SLN set.**

| **Variable** | **Model Type** | **CU** | **AUC** | **CI_l_** | **CI_u_** | **Acc** | **Sens** | **Spec** | | **PPV** | **NPV** | **R^2^** | **Y** |
| --- | --- | --- | --- | --- | --- | --- | --- | --- | --- | --- | --- | --- | --- |
| ADC (EPI) | Logistic Regression | 874 | 0,93 | 0,84 | 1 | 0,75 | 1 | | 0,72 | 0,28 | 1 | 0,47 | 0,04 |
| ADC (EPI) | Random Forest | 837 | 0,81 | 0,62 | 1 | 0,93 | 0,57 | | 0,97 | 0,67 | 0,95 |  | 0,35 |
| ADC (EPI) | XGBoost | 874 | 0,99 | 0,96 | 1 | 0,97 | 1 | | 0,97 | 0,78 | 1 |  | 0,08 |
| ADC (EPI) | KNN | 823 | 0,89 | 0,83 | 0,96 | 0,23 | 0 | | 0,25 | 0 | 0,7 |  | 0,82 |
| ADC (EPI) | Naive Bayes | 882 | 0,93 | 0,84 | 1 | 0,75 | 1 | | 0,72 | 0,28 | 1 |  | 0,06 |
| ADC (EPI) | Neural Net | 874 | 0,93 | 0,84 | 1 | 0,75 | 1 | | 0,72 | 0,28 | 1 |  | 0,09 |
| ADC (RES) | Logistic Regression | 925 | 0,85 | 0,73 | 0,97 | 0,69 | 1 | | 0,66 | 0,24 | 1 | 0,24 | 0,06 |
| ADC (RES) | Random Forest | 988 | 0,7 | 0,51 | 0,88 | 0,62 | 0,86 | | 0,59 | 0,19 | 0,97 |  | 0 |
| ADC (RES) | XGBoost | 785 | 0,99 | 0,97 | 1 | 0,96 | 1 | | 0,95 | 0,7 | 1 |  | 0,2 |
| ADC (RES) | KNN | 988 | 0,9 | 0,82 | 0,98 | 0,21 | 0 | | 0,23 | 0 | 0,68 |  | 0,92 |
| ADC (RES) | Naive Bayes | 930 | 0,86 | 0,73 | 0,98 | 0,69 | 1 | | 0,66 | 0,24 | 1 |  | 0,08 |
| ADC (RES) | Neural Net | 930 | 0,85 | 0,73 | 0,97 | 0,69 | 1 | | 0,66 | 0,24 | 1 |  | 0,11 |
| ADC (VOL) | Logistic Regression | 1111 | 0,66 | 0,43 | 0,89 | 0,61 | 0,71 | | 0,59 | 0,16 | 0,95 | 0,05 | 0,11 |
| ADC (VOL) | Random Forest | 1020 | 0,84 | 0,62 | 1 | 0,03 | 0,29 | | 0 | 0,03 | 0 |  | 0 |
| ADC (VOL) | XGBoost | 1532 | 0,98 | 0,93 | 1 | 0,87 | 1 | | 0,86 | 0,44 | 1 |  | 0,07 |
| ADC (VOL) | KNN | 977 | 0,77 | 0,66 | 0,87 | 0,38 | 0 | | 0,42 | 0 | 0,79 |  | 0,93 |
| ADC (VOL) | Naive Bayes | 1111 | 0,66 | 0,43 | 0,89 | 0,61 | 0,71 | | 0,59 | 0,16 | 0,95 |  | 0,12 |
| ADC (VOL) | Neural Net | 1111 | 0,66 | 0,43 | 0,89 | 0,61 | 0,71 | | 0,59 | 0,16 | 0,95 |  | 0,11 |
| cADC (EPI) | Logistic Regression | 0,81 | 0,75 | 0,47 | 1 | 0,92 | 0,71 | | 0,94 | 0,56 | 0,97 | 0,2 | 0,21 |
| cADC (EPI) | Random Forest | 0,76 | 0,73 | 0,53 | 0,93 | 0,87 | 0,57 | | 0,91 | 0,4 | 0,95 |  | 0,28 |
| cADC (EPI) | XGBoost | 1,13 | 0,98 | 0,96 | 1 | 0,94 | 1 | | 0,94 | 0,64 | 1 |  | 0,08 |
| cADC (EPI) | KNN | 0,93 | 0,92 | 0,85 | 0,98 | 0,21 | 0 | | 0,23 | 0 | 0,68 |  | 0,9 |
| cADC (EPI) | Naive Bayes | 0,81 | 0,75 | 0,46 | 1 | 0,92 | 0,71 | | 0,94 | 0,56 | 0,97 |  | 0,16 |
| cADC (EPI) | Neural Net | 0,81 | 0,75 | 0,47 | 1 | 0,92 | 0,71 | | 0,94 | 0,56 | 0,97 |  | 0,22 |
| cADC (RES) | Logistic Regression | 0,77 | 0,69 | 0,42 | 0,96 | 0,89 | 0,57 | | 0,92 | 0,44 | 0,95 | 0,09 | 0,17 |
| cADC (RES) | Random Forest | 0,77 | 0,47 | 0,18 | 0,75 | 0,39 | 0,43 | | 0,39 | 0,07 | 0,86 |  | 0,01 |
| cADC (RES) | XGBoost | 1,48 | 0,98 | 0,96 | 1 | 0,93 | 1 | | 0,92 | 0,58 | 1 |  | 0,07 |
| cADC (RES) | KNN | 0,9 | 0,9 | 0,83 | 0,98 | 0,28 | 0 | | 0,31 | 0 | 0,74 |  | 0,9 |
| cADC (RES) | Naive Bayes | 0,77 | 0,68 | 0,4 | 0,97 | 0,89 | 0,57 | | 0,92 | 0,44 | 0,95 |  | 0,14 |
| cADC (RES) | Neural Net | 0,77 | 0,69 | 0,42 | 0,96 | 0,89 | 0,57 | | 0,92 | 0,44 | 0,95 |  | 0,18 |
| cADC (VOL) | Logistic Regression | 0,81 | 0,62 | 0,35 | 0,88 | 0,83 | 0,43 | | 0,88 | 0,27 | 0,93 | 0,02 | 0,13 |
| cADC (VOL) | Random Forest | 0,88 | 0,63 | 0,33 | 0,94 | 0,18 | 0,43 | | 0,16 | 0,05 | 0,71 |  | 0 |
| cADC (VOL) | XGBoost | 1,39 | 0,98 | 0,93 | 1 | 0,85 | 1 | | 0,83 | 0,39 | 1 |  | 0,07 |
| cADC (VOL) | KNN | 1,06 | 0,84 | 0,75 | 0,93 | 0,37 | 0 | | 0,41 | 0 | 0,79 |  | 0,9 |
| cADC (VOL) | Naive Bayes | 0,98 | 0,65 | 0,42 | 0,87 | 0,51 | 0,86 | | 0,47 | 0,15 | 0,97 |  | 0,08 |
| cADC (VOL) | Neural Net | 0,79 | 0,62 | 0,35 | 0,88 | 0,83 | 0,43 | | 0,88 | 0,27 | 0,93 |  | 0,11 |
| mADC (EPI) | Logistic Regression | 0,49 | 0,9 | 0,78 | 1 | 0,94 | 0,71 | | 0,97 | 0,71 | 0,97 | 0,37 | 0,31 |
| mADC (EPI) | Random Forest | 0,48 | 0,74 | 0,5 | 0,97 | 0,9 | 0,57 | | 0,94 | 0,5 | 0,95 |  | 0,31 |
| mADC (EPI) | XGBoost | 0,59 | 0,99 | 0,97 | 1 | 0,96 | 1 | | 0,95 | 0,7 | 1 |  | 0,12 |
| mADC (EPI) | KNN | 0,59 | 0,93 | 0,87 | 0,99 | 0,23 | 0 | | 0,25 | 0 | 0,7 |  | 0,9 |
| mADC (EPI) | Naive Bayes | 0,49 | 0,9 | 0,78 | 1 | 0,94 | 0,71 | | 0,97 | 0,71 | 0,97 |  | 0,3 |
| mADC (EPI) | Neural Net | 0,49 | 0,9 | 0,78 | 1 | 0,94 | 0,71 | | 0,97 | 0,71 | 0,97 |  | 0,27 |
| mADC (RES) | Logistic Regression | 0,6 | 0,8 | 0,64 | 0,96 | 0,52 | 1 | | 0,47 | 0,17 | 1 | 0,18 | 0,05 |
| mADC (RES) | Random Forest | 0,51 | 0,47 | 0,2 | 0,74 | 0,41 | 0,43 | | 0,41 | 0,07 | 0,87 |  | 0,01 |
| mADC (RES) | XGBoost | 0,6 | 0,98 | 0,95 | 1 | 0,92 | 1 | | 0,91 | 0,54 | 1 |  | 0,09 |
| mADC (RES) | KNN | 0,51 | 0,85 | 0,76 | 0,94 | 0,37 | 0 | | 0,41 | 0 | 0,79 |  | 0,9 |
| mADC (RES) | Naive Bayes | 0,6 | 0,8 | 0,64 | 0,97 | 0,52 | 1 | | 0,47 | 0,17 | 1 |  | 0,05 |
| mADC (RES) | Neural Net | 0,61 | 0,8 | 0,64 | 0,96 | 0,52 | 1 | | 0,47 | 0,17 | 1 |  | 0,1 |
| mADC (VOL) | Logistic Regression | 0,74 | 0,62 | 0,41 | 0,83 | 0,58 | 0,71 | | 0,56 | 0,15 | 0,95 | 0,04 | 0,11 |
| mADC (VOL) | Random Forest | 0,66 | 0,89 | 0,75 | 1 | 0,03 | 0,29 | | 0 | 0,03 | 0 |  | 0 |
| mADC (VOL) | XGBoost | 0,69 | 0,96 | 0,89 | 1 | 0,8 | 1 | | 0,78 | 0,33 | 1 |  | 0,09 |
| mADC (VOL) | KNN | 0,67 | 0,84 | 0,78 | 0,91 | 0,27 | 0 | | 0,3 | 0 | 0,73 |  | 0,9 |
| mADC (VOL) | Naive Bayes | 0,74 | 0,61 | 0,41 | 0,82 | 0,58 | 0,71 | | 0,56 | 0,15 | 0,95 |  | 0,12 |
| mADC (VOL) | Neural Net | 0,74 | 0,62 | 0,41 | 0,83 | 0,58 | 0,71 | | 0,56 | 0,15 | 0,95 |  | 0,11 |
| SAD | Logistic Regression | 6 | 0,61 | 0,38 | 0,84 | 0,54 | 0,71 | | 0,52 | 0,14 | 0,94 | 0,01 | 0,09 |
| SAD | Random Forest | 10 | 0,82 | 0,66 | 0,97 | 0,14 | 0,29 | | 0,13 | 0,03 | 0,62 |  | 0,01 |
| SAD | XGBoost | 6 | 0,68 | 0,48 | 0,88 | 0,61 | 0,71 | | 0,59 | 0,16 | 0,95 |  | 0,1 |
| SAD | KNN | 6 | 0,7 | 0,51 | 0,89 | 0,49 | 0,14 | | 0,53 | 0,03 | 0,85 |  | 0,89 |
| SAD | Naive Bayes | 5 | 0,61 | 0,38 | 0,84 | 0,54 | 0,71 | | 0,52 | 0,14 | 0,94 |  | 0,08 |
| SAD | Neural Net | 6 | 0,61 | 0,38 | 0,84 | 0,54 | 0,71 | | 0,52 | 0,14 | 0,94 |  | 0,1 |
| Node-RADS | Logistic Regression | 5 | 0,5 | 0,27 | 0,72 | 0,92 | 0,14 | | 1 | 1 | 0,91 | 0,01 | 0,16 |
| Node-RADS | Random Forest | 1 | 0,57 | 0,36 | 0,79 | 0,27 | 0,43 | | 0,25 | 0,06 | 0,8 |  | 0,08 |
| Node-RADS | XGBoost | 2 | 0,59 | 0,37 | 0,8 | 0,68 | 0,43 | | 0,7 | 0,14 | 0,92 |  | 0,11 |
| Node-RADS | KNN | 3 | 0,4 | 0,2 | 0,59 | 0,1 | 1 | | 0 | 0,1 | xx |  | xx |
| Node-RADS | Naive Bayes | 4 | 0,43 | 0,19 | 0,67 | 0,92 | 0,14 | | 1 | 1 | 0,91 |  | 0,64 |
| Node-RADS | Neural Net | 4 | 0,5 | 0,27 | 0,72 | 0,92 | 0,14 | | 1 | 1 | 0,91 |  | 0,14 |

ADC Apparent diffusion coefficient in × 10⁻⁶ mm²/s, AUC Area under the curve, Acc Accuracy, cADC Ratio of SLN ADC to mean ADC of 3 contralateral LNs, CI_l_ Lower confidence interval, CI_u_ Upper confidence interval, *CU* cut-off EPI Single shot spinecho echoplanar imaging, KNN K-Nearest Neighbors, mADC Ratio of SLN ADC to ADC of ipsilateral muscle tissue, Node-RADS Node Reporting and Data System, NPV Negative predictive value, PPV Positive predictive value, RES Readout segmentation of long variable echo trains sequence (RESOLVE), R^2^ McFadden Index, SAD Short axis diameter in mm, Sens Sensitivity, SLNE Sentinel lymph node extraction, Spec Specificity, VOL Volumetry measuring, XGBoost Gradient Boosting.

**Supplementary Tab. 7** Model validation using machine learning algorithms: performance measures for MLN set.

| **Variable** | **Model Type** | **CU** | **AUC** | **CI_l_** | **CI_u_** | **Acc** | **Sens** | **Spec** | **PPV** | **NPV** | **R^2^** | **Y** |
| --- | --- | --- | --- | --- | --- | --- | --- | --- | --- | --- | --- | --- |
| ADC (EPI) | Logistic Regression | 781 | 0,99 | 0,98 | 1 | 0,95 | 0,99 | 0,91 | 0,93 | 0,98 | 0,83 | 0,35 |
| ADC (EPI) | Random Forest | 820 | 0,98 | 0,95 | 1 | 0,05 | 0 | 0,1 | 0 | 0,09 |  | 0,86 |
| ADC (EPI) | XGBoost | 783 | 1 | 1 | 1 | 0,99 | 0,99 | 0,99 | 0,99 | 0,99 |  | 0,5 |
| ADC (EPI) | KNN | 732 | 0,45 | 0,4 | 0,51 | 0,53 | 0,96 | 0,06 | 0,53 | 0,57 |  | 0,7 |
| ADC (EPI) | Naive Bayes | 780 | 0,99 | 0,98 | 1 | 0,95 | 0,99 | 0,91 | 0,93 | 0,98 |  | 0,4 |
| ADC (EPI) | Neural Net | 780 | 0,99 | 0,98 | 1 | 0,95 | 0,99 | 0,91 | 0,93 | 0,98 |  | 0,43 |
| ADC (RES) | Logistic Regression | 809 | 0,83 | 0,76 | 0,9 | 0,76 | 0,65 | 0,88 | 0,86 | 0,7 | 0,23 | 0,66 |
| ADC (RES) | Random Forest | 930 | 0,76 | 0,68 | 0,84 | 0,28 | 0,24 | 0,33 | 0,28 | 0,29 |  | 0,56 |
| ADC (RES) | XGBoost | 1219 | 0,99 | 0,97 | 1 | 0,97 | 0,97 | 0,96 | 0,96 | 0,97 |  | 0,48 |
| ADC (RES) | KNN | 764 | 0,55 | 0,46 | 0,64 | 0,58 | 0,44 | 0,73 | 0,64 | 0,54 |  | 0,82 |
| ADC (RES) | Naive Bayes | 809 | 0,83 | 0,76 | 0,9 | 0,76 | 0,65 | 0,88 | 0,86 | 0,7 |  | 0,69 |
| ADC (RES) | Neural Net | 809 | 0,83 | 0,76 | 0,9 | 0,76 | 0,65 | 0,88 | 0,86 | 0,7 |  | 0,62 |
| ADC (VOL) | Logistic Regression | 850 | 0,97 | 0,94 | 0,99 | 0,9 | 0,85 | 0,94 | 0,94 | 0,86 | 0,66 | 0,77 |
| ADC (VOL) | Random Forest | 980 | 0,93 | 0,9 | 0,97 | 0,14 | 0,03 | 0,26 | 0,04 | 0,2 |  | 0,85 |
| ADC (VOL) | XGBoost | 980 | 0,99 | 0,98 | 1 | 0,97 | 0,96 | 0,97 | 0,97 | 0,96 |  | 0,63 |
| ADC (VOL) | KNN | 816 | 0,49 | 0,41 | 0,56 | 0,53 | 0,85 | 0,19 | 0,53 | 0,54 |  | 0,63 |
| ADC (VOL) | Naive Bayes | 945 | 0,96 | 0,93 | 0,99 | 0,9 | 0,95 | 0,84 | 0,87 | 0,94 |  | 0,45 |
| ADC (VOL) | Neural Net | 856 | 0,97 | 0,94 | 0,99 | 0,9 | 0,85 | 0,94 | 0,94 | 0,86 |  | 0,72 |
| cADC (EPI) | Logistic Regression | 0,83 | 0,98 | 0,96 | 1 | 0,94 | 0,99 | 0,9 | 0,91 | 0,98 | 0,74 | 0,39 |
| cADC (EPI) | Random Forest | 0,82 | 0,98 | 0,96 | 1 | 0,06 | 0,03 | 0,1 | 0,03 | 0,09 |  | 0,61 |
| cADC (EPI) | XGBoost | 0,81 | 1 | 0,99 | 1 | 0,99 | 0,99 | 0,99 | 0,99 | 0,99 |  | 0,51 |
| cADC (EPI) | KNN | 0,61 | 0,53 | 0,46 | 0,59 | 0,54 | 0,83 | 0,23 | 0,54 | 0,55 |  | 0,9 |
| cADC (EPI) | Naive Bayes | 0,83 | 0,98 | 0,96 | 1 | 0,94 | 0,99 | 0,9 | 0,91 | 0,98 |  | 0,43 |
| cADC (EPI) | Neural Net | 0,84 | 0,98 | 0,96 | 1 | 0,94 | 0,99 | 0,9 | 0,91 | 0,98 |  | 0,46 |
| cADC (RES) | Logistic Regression | 0,8 | 0,83 | 0,77 | 0,9 | 0,76 | 0,63 | 0,91 | 0,89 | 0,69 | 0,26 | 0,61 |
| cADC (RES) | Random Forest | 0,57 | 0,75 | 0,66 | 0,83 | 0,28 | 0,55 | 0 | 0,37 | 0 |  | 0,03 |
| cADC (RES) | XGBoost | 1,1 | 1 | 0,99 | 1 | 0,98 | 0,97 | 0,99 | 0,99 | 0,97 |  | 0,49 |
| cADC (RES) | KNN | 0,57 | 0,6 | 0,51 | 0,69 | 0,64 | 0,48 | 0,81 | 0,74 | 0,59 |  | 0,9 |
| cADC (RES) | Naive Bayes | 0,79 | 0,83 | 0,77 | 0,9 | 0,76 | 0,63 | 0,91 | 0,89 | 0,69 |  | 0,59 |
| cADC (RES) | Neural Net | 0,8 | 0,83 | 0,77 | 0,9 | 0,76 | 0,63 | 0,91 | 0,89 | 0,69 |  | 0,56 |
| cADC (VOL) | Logistic Regression | 0,84 | 0,91 | 0,86 | 0,96 | 0,85 | 0,88 | 0,81 | 0,84 | 0,86 | 0,44 | 0,47 |
| cADC (VOL) | Random Forest | 1,03 | 0,86 | 0,8 | 0,92 | 0,19 | 0,2 | 0,19 | 0,21 | 0,18 |  | 0,5 |
| cADC (VOL) | XGBoost | 1,04 | 0,99 | 0,98 | 1 | 0,99 | 0,99 | 0,99 | 0,99 | 0,99 |  | 0,53 |
| cADC (VOL) | KNN | 0,7 | 0,55 | 0,46 | 0,64 | 0,54 | 0,52 | 0,57 | 0,57 | 0,52 |  | 0,9 |
| cADC (VOL) | Naive Bayes | 0,84 | 0,91 | 0,86 | 0,96 | 0,85 | 0,88 | 0,81 | 0,84 | 0,86 |  | 0,5 |
| cADC (VOL) | Neural Net | 0,83 | 0,91 | 0,86 | 0,96 | 0,85 | 0,88 | 0,81 | 0,84 | 0,86 |  | 0,46 |
| mADC (EPI) | Logistic Regression | 0,49 | 0,99 | 0,98 | 1 | 0,96 | 0,95 | 0,97 | 0,97 | 0,94 | 0,82 | 0,66 |
| mADC (EPI) | Random Forest | 0,49 | 0,99 | 0,99 | 1 | 0,04 | 0,08 | 0 | 0,08 | 0 |  | 0,19 |
| mADC (EPI) | XGBoost | 0,52 | 1 | 1 | 1 | 0,99 | 1 | 0,99 | 0,99 | 1 |  | 0,45 |
| mADC (EPI) | KNN | 0,4 | 0,49 | 0,43 | 0,55 | 0,52 | 1 | 0 | 0,52 | xx |  | xx |
| mADC (EPI) | Naive Bayes | 0,49 | 0,99 | 0,98 | 1 | 0,96 | 0,95 | 0,97 | 0,97 | 0,94 |  | 0,67 |
| mADC (EPI) | Neural Net | 0,49 | 0,99 | 0,98 | 1 | 0,96 | 0,95 | 0,97 | 0,97 | 0,94 |  | 0,62 |
| mADC (RES) | Logistic Regression | 0,51 | 0,85 | 0,78 | 0,91 | 0,79 | 0,72 | 0,87 | 0,86 | 0,74 | 0,27 | 0,62 |
| mADC (RES) | Random Forest | 0,51 | 0,79 | 0,72 | 0,86 | 0,27 | 0,25 | 0,29 | 0,28 | 0,26 |  | 0,51 |
| mADC (RES) | XGBoost | 0,59 | 0,99 | 0,98 | 1 | 0,99 | 0,99 | 0,99 | 0,99 | 0,99 |  | 0,5 |
| mADC (RES) | KNN | 0,41 | 0,54 | 0,46 | 0,63 | 0,56 | 0,43 | 0,7 | 0,6 | 0,53 |  | 0,9 |
| mADC (RES) | Naive Bayes | 0,51 | 0,85 | 0,78 | 0,91 | 0,79 | 0,72 | 0,87 | 0,86 | 0,74 |  | 0,67 |
| mADC (RES) | Neural Net | 0,51 | 0,85 | 0,78 | 0,91 | 0,79 | 0,72 | 0,87 | 0,86 | 0,74 |  | 0,58 |
| mADC (VOL) | Logistic Regression | 0,57 | 0,96 | 0,93 | 0,98 | 0,88 | 0,84 | 0,93 | 0,93 | 0,84 | 0,6 | 0,71 |
| mADC (VOL) | Random Forest | 0,5 | 0,93 | 0,89 | 0,97 | 0,11 | 0,05 | 0,17 | 0,07 | 0,15 |  | 0,68 |
| mADC (VOL) | XGBoost | 0,54 | 0,99 | 0,98 | 1 | 0,99 | 0,99 | 0,99 | 0,99 | 0,99 |  | 0,52 |
| mADC (VOL) | KNN | 0,47 | 0,49 | 0,41 | 0,57 | 0,52 | 1 | 0 | 0,52 | xx |  | xx |
| mADC (VOL) | Naive Bayes | 0,64 | 0,95 | 0,92 | 0,98 | 0,88 | 0,93 | 0,81 | 0,84 | 0,92 |  | 0,52 |
| mADC (VOL) | Neural Net | 0,57 | 0,96 | 0,93 | 0,98 | 0,88 | 0,84 | 0,93 | 0,93 | 0,84 |  | 0,66 |
| SAD | Logistic Regression | 7 | 0,86 | 0,8 | 0,92 | 0,76 | 0,75 | 0,78 | 0,79 | 0,74 | 0,34 | 0,48 |
| SAD | Random Forest | 10 | 0,86 | 0,8 | 0,92 | 0,19 | 0,11 | 0,28 | 0,14 | 0,22 |  | 0,61 |
| SAD | XGBoost | 8 | 0,91 | 0,86 | 0,95 | 0,81 | 0,85 | 0,77 | 0,8 | 0,83 |  | 0,54 |
| SAD | KNN | 4 | 0,53 | 0,44 | 0,62 | 0,44 | 0,41 | 0,48 | 0,46 | 0,43 |  | 0,87 |
| SAD | Naive Bayes | 9 | 0,79 | 0,72 | 0,87 | 0,75 | 0,64 | 0,87 | 0,84 | 0,69 |  | 0,38 |
| SAD | Neural Net | 7 | 0,86 | 0,8 | 0,92 | 0,76 | 0,75 | 0,78 | 0,79 | 0,74 |  | 0,56 |
| Node-RADS | Logistic Regression | 3 | 0,89 | 0,84 | 0,94 | 0,81 | 0,87 | 0,74 | 0,78 | 0,84 | 0,42 | 0,44 |
| Node-RADS | Random Forest | 3 | 0,82 | 0,75 | 0,89 | 0,19 | 0,13 | 0,26 | 0,16 | 0,22 |  | 0,57 |
| Node-RADS | XGBoost | 3 | 0,89 | 0,84 | 0,94 | 0,81 | 0,87 | 0,74 | 0,78 | 0,84 |  | 0,44 |
| Node-RADS | KNN | 5 | 0,59 | 0,5 | 0,69 | 0,65 | 0,33 | 1 | 1 | 0,58 |  | 0,97 |
| Node-RADS | Naive Bayes | 2 | 0,89 | 0,84 | 0,94 | 0,81 | 0,87 | 0,74 | 0,78 | 0,84 |  | 0,42 |
| Node-RADS | Neural Net | 2 | 0,89 | 0,84 | 0,94 | 0,81 | 0,87 | 0,74 | 0,78 | 0,84 |  | 0,43 |

Acc Accuracy, AUC Area under the curve, ADC Apparent diffusion coefficient in × 10⁻⁶ mm²/s, CI_l_ Lower confidence interval, CI_u_ Upper confidence interval, cADC Ratio of SLN ADC to mean ADC of 3 contralateral LNs, CU Cut-off, EPI Single shot spinecho echoplanar imaging, KNN K-Nearest Neighbors, mADC Ratio of SLN ADC to ADC of ipsilateral muscle tissue, MLN Confirmed metastases from tumor board, Node-RADS Node Reporting and Data System, NPV Negative predictive value, PPV Positive predictive value, RES Readout segmentation of long variable echo trains sequence (RESOLVE), R^2^ McFadden Index, SAD Short axis diameter in mm, Sens Sensitivity, Spec Specificity, VOL Volumetry measuring, XGBoost Gradient Boosting,

**Supplementary Tab. 8** Results from reliability estimation using Gwet’s AC1 coefficient based on optimal cut-offs from logistic regression in SLN set and MLN set.

|  |  | SLNE | |  |  |  | MLNpooled | | |  |
| --- | --- | --- | --- | --- | --- | --- | --- | --- | --- | --- |
| **Model** | **Variable** | **CU** | **EST** | **CI_l_** | **CI_u_** |  | **CU** | **EST** | **CI_l_** | **CI_u_** |
| Logistic Regression | ADC (EPI) | 874 | 0,59 | 0,38 | 0,76 |  | 781 | 0,9 | 0,82 | 0,96 |
| Random Forest | ADC (EPI) | 837 | 0,79 | 0,64 | 0,91 |  | 820 | 0,9 | 0,83 | 0,97 |
| XGBoost | ADC (EPI) | 874 | 0,59 | 0,4 | 0,77 |  | 783 | 0,89 | 0,81 | 0,96 |
| KNN | ADC (EPI) | 823 | 0,81 | 0,68 | 0,92 |  | 732 | 0,88 | 0,79 | 0,95 |
| Naive Bayes | ADC (EPI) | 882 | 0,61 | 0,42 | 0,79 |  | 780 | 0,89 | 0,82 | 0,96 |
| Neural Net | ADC (EPI) | 874 | 0,59 | 0,39 | 0,77 |  | 780 | 0,89 | 0,81 | 0,96 |
| Logistic Regression | ADC (RES) | 925 | 0,49 | 0,26 | 0,69 |  | 809 | 0,52 | 0,38 | 0,64 |
| Random Forest | ADC (RES) | 988 | 0,2 | -0,04 | 0,42 |  | 930 | 0,53 | 0,39 | 0,67 |
| XGBoost | ADC (RES) | 785 | 0,82 | 0,7 | 0,92 |  | 1219 | 0,28 | 0,11 | 0,46 |
| KNN | ADC (RES) | 988 | 0,2 | -0,07 | 0,44 |  | 764 | 0,48 | 0,33 | 0,63 |
| Naive Bayes | ADC (RES) | 930 | 0,5 | 0,25 | 0,71 |  | 809 | 0,52 | 0,38 | 0,66 |
| Neural Net | ADC (RES) | 930 | 0,5 | 0,28 | 0,7 |  | 809 | 0,52 | 0,37 | 0,66 |
| Logistic Regression | ADC (VOL) | 1111 | 0,35 | 0,1 | 0,56 |  | 850 | 0,78 | 0,67 | 0,88 |
| Random Forest | ADC (VOL) | 1020 | 0,47 | 0,24 | 0,66 |  | 980 | 0,77 | 0,66 | 0,87 |
| XGBoost | ADC (VOL) | 1532 | -0,45 | -0,66 | -0,2 |  | 980 | 0,77 | 0,65 | 0,86 |
| KNN | ADC (VOL) | 977 | 0,7 | 0,51 | 0,84 |  | 816 | 0,71 | 0,6 | 0,82 |
| Naive Bayes | ADC (VOL) | 1111 | 0,35 | 0,1 | 0,56 |  | 945 | 0,78 | 0,68 | 0,88 |
| Neural Net | ADC (VOL) | 1111 | 0,35 | 0,12 | 0,57 |  | 856 | 0,79 | 0,68 | 0,89 |
| Logistic Regression | cADC (EPI) | 0,81 | 0,88 | 0,77 | 0,96 |  | 0,83 | 0,89 | 0,81 | 0,96 |
| Random Forest | cADC (EPI) | 0,76 | 0,88 | 0,78 | 0,96 |  | 0,82 | 0,88 | 0,79 | 0,95 |
| XGBoost | cADC (EPI) | 1,13 | -0,58 | -0,75 | -0,4 |  | 0,81 | 0,86 | 0,78 | 0,94 |
| KNN | cADC (EPI) | 0,93 | 0,55 | 0,31 | 0,73 |  | 0,61 | 0,44 | 0,29 | 0,58 |
| Naive Bayes | cADC (EPI) | 0,81 | 0,88 | 0,78 | 0,95 |  | 0,83 | 0,89 | 0,81 | 0,96 |
| Neural Net | cADC (EPI) | 0,81 | 0,88 | 0,77 | 0,96 |  | 0,84 | 0,88 | 0,79 | 0,96 |
| Logistic Regression | cADC (RES) | 0,77 | 0,84 | 0,73 | 0,93 |  | 0,8 | 0,52 | 0,38 | 0,65 |
| Random Forest | cADC (RES) | 0,77 | 0,84 | 0,73 | 0,94 |  | 0,57 | 0,36 | 0,17 | 0,51 |
| XGBoost | cADC (RES) | 1,48 | -0,79 | -0,91 | -0,6 |  | 1,1 | 0,33 | 0,16 | 0,49 |
| KNN | cADC (RES) | 0,9 | 0,24 | -0,02 | 0,47 |  | 0,57 | 0,36 | 0,19 | 0,51 |
| Naive Bayes | cADC (RES) | 0,77 | 0,84 | 0,72 | 0,94 |  | 0,79 | 0,52 | 0,38 | 0,66 |
| Neural Net | cADC (RES) | 0,77 | 0,84 | 0,71 | 0,94 |  | 0,8 | 0,52 | 0,38 | 0,66 |
| Logistic Regression | cADC (VOL) | 0,81 | -0,01 | -0,06 | 0 |  | 0,84 | 0 | 0 | -0,01 |
| Random Forest | cADC (VOL) | 0,88 | -0,01 | -0,06 | 0 |  | 1,03 | 0 | 0 | -0,01 |
| XGBoost | cADC (VOL) | 1,39 | -0,01 | -0,06 | 0 |  | 1,04 | 0 | 0 | -0,01 |
| KNN | cADC (VOL) | 1,06 | -0,01 | -0,06 | 0 |  | 0,7 | 0 | 0 | -0,01 |
| Naive Bayes | cADC (VOL) | 0,98 | -0,01 | -0,06 | 0 |  | 0,84 | 0 | 0 | -0,01 |
| Neural Net | cADC (VOL) | 0,79 | -0,01 | -0,06 | 0 |  | 0,83 | 0 | 0 | -0,01 |
| Logistic Regression | mADC (EPI) | 0,49 | 0,92 | 0,83 | 0,98 |  | 0,49 | 0,92 | 0,85 | 0,97 |
| Random Forest | mADC (EPI) | 0,48 | 0,92 | 0,83 | 0,98 |  | 0,49 | 0,92 | 0,85 | 0,97 |
| XGBoost | mADC (EPI) | 0,59 | 0,47 | 0,26 | 0,66 |  | 0,52 | 0,89 | 0,81 | 0,96 |
| KNN | mADC (EPI) | 0,59 | 0,44 | 0,2 | 0,66 |  | 0,4 | 0,44 | 0,29 | 0,58 |
| Naive Bayes | mADC (EPI) | 0,49 | 0,91 | 0,83 | 0,98 |  | 0,49 | 0,9 | 0,83 | 0,97 |
| Neural Net | mADC (EPI) | 0,49 | 0,91 | 0,82 | 0,98 |  | 0,49 | 0,92 | 0,85 | 0,97 |
| Logistic Regression | mADC (RES) | 0,6 | 0,13 | -0,12 | 0,39 |  | 0,51 | 0,57 | 0,43 | 0,71 |
| Random Forest | mADC (RES) | 0,51 | 0,71 | 0,54 | 0,85 |  | 0,51 | 0,56 | 0,42 | 0,7 |
| XGBoost | mADC (RES) | 0,6 | 0,13 | -0,14 | 0,37 |  | 0,59 | 0,45 | 0,3 | 0,61 |
| KNN | mADC (RES) | 0,51 | 0,71 | 0,54 | 0,85 |  | 0,41 | 0,29 | 0,11 | 0,44 |
| Naive Bayes | mADC (RES) | 0,6 | 0,13 | -0,12 | 0,39 |  | 0,51 | 0,57 | 0,43 | 0,71 |
| Neural Net | mADC (RES) | 0,61 | 0,1 | -0,16 | 0,35 |  | 0,51 | 0,57 | 0,45 | 0,7 |
| Logistic Regression | mADC (VOL) | 0,74 | 0,26 | 0 | 0,51 |  | 0,57 | 0,76 | 0,65 | 0,86 |
| Random Forest | mADC (VOL) | 0,66 | 0,53 | 0,32 | 0,71 |  | 0,5 | 0,48 | 0,34 | 0,63 |
| XGBoost | mADC (VOL) | 0,69 | 0,35 | 0,1 | 0,57 |  | 0,54 | 0,67 | 0,54 | 0,79 |
| KNN | mADC (VOL) | 0,67 | 0,52 | 0,31 | 0,7 |  | 0,47 | 0,35 | 0,18 | 0,5 |
| Naive Bayes | mADC (VOL) | 0,74 | 0,26 | 0,02 | 0,51 |  | 0,64 | 0,75 | 0,64 | 0,86 |
| Neural Net | mADC (VOL) | 0,74 | 0,26 | -0,01 | 0,51 |  | 0,57 | 0,75 | 0,64 | 0,86 |
| Logistic Regression | SAD | 6 | 0,42 | 0,18 | 0,65 |  | 7 | 0,53 | 0,39 | 0,67 |
| Random Forest | SAD | 10 | 0,83 | 0,7 | 0,92 |  | 10 | 0,4 | 0,26 | 0,55 |
| XGBoost | SAD | 6 | 0,42 | 0,16 | 0,64 |  | 8 | 0,5 | 0,35 | 0,65 |
| KNN | SAD | 6 | 0,42 | 0,18 | 0,64 |  | 4 | 0,39 | 0,22 | 0,54 |
| Naive Bayes | SAD | 5 | 0,2 | -0,05 | 0,45 |  | 9 | 0,39 | 0,24 | 0,54 |
| Neural Net | SAD | 6 | 0,42 | 0,2 | 0,64 |  | 7 | 0,53 | 0,38 | 0,67 |
| Logistic Regression | Node-RADS | 5 | 0,89 | 0,8 | 0,96 |  | 3 | 0,52 | 0,38 | 0,66 |
| Random Forest | Node-RADS | 1 | 0,05 | -0,23 | 0,34 |  | 3 | 0,52 | 0,37 | 0,66 |
| XGBoost | Node-RADS | 2 | 0,57 | 0,35 | 0,74 |  | 3 | 0,52 | 0,37 | 0,66 |
| KNN | Node-RADS | 3 | 0,89 | 0,79 | 0,97 |  | 5 | 0,15 | 0 | 0,34 |
| Naive Bayes | Node-RADS | 4 | 0,91 | 0,81 | 0,97 |  | 2 | 0,62 | 0,49 | 0,75 |
| Neural Net | Node-RADS | 4 | 0,91 | 0,82 | 0,97 |  | 2 | 0,62 | 0,49 | 0,74 |

ADC Apparent diffusion coefficient in × 10⁻⁶ mm²/s, cADC Ratio of SLN ADC to mean ADC of 3 contralateral LNs, CI_l_ Lower confidence interval, CI_u_ Upper confidence interval, CU Cut-off, EST Estimation, EPI Single shot spinecho echoplanar imaging, KNN K-Nearest Neighbors, MLN Confirmed metastasized lymph nodes, Node RADS Node Reporting and Data System, RES Readout segmentation of long variable echo trains sequence (RESOLVE), R^2^ McFadden Index, SAD Short axis diameter in mm, SLN Sentinel lymph node, VOL Volumetry measuring, XGBoost Gradient Boosting.

***Formulas 1*** *Definition of the metrics in the confusion’s matrix*

$$TP=\sum_{i=1}^{n} \boldsymbol{1}(Sp_{i}=1 and\text{ }St_{i}=\text{diseased})$$

$$FP=\sum_{i=1}^{n} \boldsymbol{1}(Sp_{i}=1\text{ }and\text{ }St_{i}=\text{non-diseased})$$

$$FN=\sum_{i=1}^{n} \boldsymbol{1}(Sp_{i}=0 and\text{ }St_{i}=\text{diseased})$$

$$TN=\sum_{i=1}^{n} \boldsymbol{1}(Sp_{i}=0 and\text{ }St_{i}=\text{non-diseased})$$

*With Sp= predicted status and St= true status*

**Supplementary Text 1: *Hyperparameters for Machine Learning Models***

All ML models were implemented in R using default settings of the respective libraries, with only minimal explicit specifications (XGBoost: logistic objective and 100 boosting rounds; KNN: k = 5; Neural Network: size = 5, decay = 0.01, maxit = 1000), while all remaining hyperparameters remained at their defaults:

**Random Forest**

call: Ranger::ranger

parameters: num.trees = 500, mtry = √p, min.node.size = 1, sample.fraction = 1.0, replace = TRUE, probability = FALSE (set to TRUE), splitrule = "gini", importance = "none", write.forest = TRUE, respect.unordered.factors = "ignore", max.depth = NULL, seed = NULL, num.threads = 1, classification = automatisch basierend auf Datentyp, verbose = FALSE

**Gradient Boosting**

call: xgboost::xgboost

parameters: eta = 0.3, gamma = 0, max_depth = 6, min_child_weight = 1, subsample = 1, colsample_bytree = 1, colsample_bylevel = 1, colsample_bynode = 1, lambda = 1, alpha = 0, scale_pos_weight = 1, max_delta_step = 0, tree_method = "auto", grow_policy = "depthwise", max_leaves = 0, max_bin = 256, monotone_constraints = NULL, objective = "binary:logistic", eval_metric = "logloss", base_score = 0.5, num_class = NULL, nrounds = 100, nthread = NULL, verbose = 1, early_stopping_rounds = NULL, missing = NaN, n_estimators = NULL, max_cat_to_onehot = NULL, enable_categorical = FALSE

**K-Nearest Neighbours**

call: class::knn

parameters: k = 5, prob = TRUE, use.all = TRUE

**Naive Bayes**

call: e1071::naiveBayes

parameters: na.action = na.pass, laplace = 0

**Neural Net**

call: nnet::nnet

parameters: weights = NULL, size = 5, Wts = NULL, mask = NULL, linout = FALSE, entropy = FALSE, softmax = FALSE, censored = FALSE, skip = FALSE, rang = 0.7, decay = 0.01, maxit = 1000, Hess = FALSE, trace = TRUE, MaxNWts = 1000, abstol = 1e-4, reltol = 1e-8, subset = NULL, na.action = na.fail, contrasts = NULL

**Supplementary Text 2** sample size calculation

For our primary set (SLN set) we performed a precision-based simulation using bootstrap confidence intervals for the statistical measures “observer agreement” and “Cohen’s kappa”. The expected widths of the 95% confidence intervals were estimated for different levels of agreement (mild: κ = 0.3; moderate: κ = 0.5; strong: κ = 0.8) and for conservative and optimistic scenarios corresponding to half-year and one-year recruitment periods. Narrower confidence intervals indicate higher precision and thus stronger statistical reliability. The assumptions for the simulation were based on observations from clinical routine, indicating an average recruitment of approximately 48 - 72 patients per half-year. In addition, more than one SLN per patient can be expected. According to the literature [1], approximately 40% of patients present with 1 SLN, about 30% with 2 SLNs, around 15% with 3 SLNs, and roughly 5% with more than 3 SLNs. This corresponds to an estimated volume of 79 to 118 SLNs per half-year (48 or 72* (0.05 * 4 + 0.15 * 3 + 0.3 * 2 + 0.4))

Assuming a consent rate of 90%, a minimum of 71 SLNs (79 × 0.9) can be expected over the course of half a calendar year (conservative scenario), increasing up to 118 SLNs when assuming a consent rate of 100% (optimistic scenario).

These estimates were used to determine the expected width of the bootstrap confidence intervals for the evaluated statistical measures.

|  | **Cohen's Kappa** | | | | **Observer agreement** | | | |
| --- | --- | --- | --- | --- | --- | --- | --- | --- |
|  | conservative | | optimistic | | conservative | | optimistic | |
|  | 1/2 year | 1 year | 1/2 year | 1 year | 1/2 year | 1 year | 1/2 year | 1 year |
|  | N=71 | N=142 | N=118 | 236 | 71 | 142 | 118 | 236 |
| **Kappa mild** (0.3) | 0,2954 | 0,2348 | 0,2574 | 0,1666 | 0,2113 | 0,1605 | 0,1681 | 0,1223 |
| **Kappa moderate** (0.5) | 0,3522 | 0,2297 | 0,2701 | 0,1885 | 0,2113 | 0,1467 | 0,1513 | 0,1054 |
| **Kappa strong** (0.8) | 0,2974 | 0,1892 | 0,2149 | 0,1437 | 0,1268 | 0,0979 | 0,1008 | 0,076 |

To reach a total of 71 SLNs, data acquisition required slightly more than one year due, among other factors, to an exclusion rate of 18 out of 70 patients.

References

1. Kunte C, Geimer T, Baumert J et al. (2010) Prognostic factors associated with sentinel lymph node positivity and effect of sentinel status on survival: an analysis of 1049 patients with cutaneous melanoma. Melanoma Res 20:330–337. https://doi.org/10.1097/CMR.0b013e32833ba9ff
